# Piglets vocally express the anticipation of pseudo-social contexts in their grunts

**DOI:** 10.1038/s41598-020-75378-x

**Published:** 2020-10-28

**Authors:** A. S. Villain, A. Hazard, M. Danglot, C. Guérin, A. Boissy, C. Tallet

**Affiliations:** 1 PEGASE, INRAE, Institut Agro, 35590 Saint Gilles, France; 2 UMRH, INRAE, VétoAgroSup, Université Clermont Auvergne, 63122 St-Genès Champanelle, France

**Keywords:** Animal behaviour, Emotion

## Abstract

Emotions not only arise in reaction to an event but also while anticipating it, making this context a means of accessing the emotional value of events. Before now, anticipatory studies have rarely considered whether vocalisations carry information about emotional states. We studied both the grunts of piglets and their spatial behaviour as they anticipated two (pseudo)social events known to elicit positive emotions of different intensity: arrival of familiar conspecifics and arrival of a familiar human. Piglets spatially anticipated both pseudo-social contexts, and the spectro temporal features of grunts differed according to the emotional context. Piglets produced low-frequency grunts at a higher rate when anticipating conspecifics compared to anticipating a human. Spectral noise increased when piglets expected conspecifics, whereas the duration and frequency range increased when expecting a human. When the arrival of conspecifics was delayed, the grunt duration increased, whereas when the arrival of the human was delayed, the spectral parameters were comparable to those during isolation. This shows that vocal expressions in piglets during anticipation are specific to the expected reward. Vocal expressions—both their temporal and spectral features- are thus a good way to explore the emotional state of piglets during the anticipation of challenging events.

## Introduction

The motivational system is one of the drivers of animal behaviour^1^. Animal emotions are important feedback mechanisms for modulating the activity of this system. One way to assess the emotional value of an event is to measure the anticipatory activity before the event. Indeed, emotions not only arise in reaction to the challenging event, but also during anticipation of this expected event^2^. Anticipation is goal directed and occurs during the appetitive phase of behaviour^3^, before the consummatory phase. Anticipatory behaviour towards a positive event is adaptive since it is associated with the motivational system that directs the animal from an aversive state (e.g., hungry) to a reinforcing state (e.g., food acquisition; see Spruijt et al.^1^). It is suggested that promoting positive anticipation is a way to enhance the quality of life of animals that are under the responsibility of humans: since anticipation may amplify emotional states, regularly inducing anticipation of positive states may promote positive emotional states and thus improve animal welfare^4^.

During anticipation of a positive event, animals are motivated for the event that will arise, and are thus more likely to pay attention to stimuli that are signalling the event itself^3^. Anticipation has been used to evaluate the degree to which different events, such as food reward, social contact or play, are positively rewarding or motivating^3,5–7^. It may also be used to evaluate the cognitive judgement bias that follows a long-term emotional experience^8^.

Anticipation of a positive situation (i.e. social contact and play) is expressed by an increase of time spent in a compartment where the given social reward is expected to arrive in rats^5^, and by an increase of activity in rats, silver foxes, pigs, horses and lambs^5,7,9–11^. Comparing the behavioural anticipation responses to different events allows for the evaluation of both the relative valence and the intensity of the emotion associated with the expected events. For instance, lambs express a higher amount of activity and more behavioural transitions before food rather than before play; the authors suggested that the food reward is a more intensely positive event^7^. In hens, behavioural anticipation is different according to the quality of the food reward^6^. In other cases, the increase of the level of activity is not specific to the type of anticipated event. For instance, in a study in pigs, the level of locomotor activity during anticipation of positive (e.g., food) and negative (e.g., frightening ) events do not differ^12^. In silver foxes, differences in anticipatory behaviour before different food rewards are shown in the posture of the ears, but not in the level of activity^10^. In goats, a higher level of activity and heart rate and a more forward ear posture were specific to anticipating a positive food reward and did not occur in a neutral or negative situation^13^. This suggests anticipatory postural and spatial behaviours may be event-specific and species-specific^14^. Due to the limited number of parameters that can be reliably monitored from one species to the other or from one context to the other, studying animal spatial and/or postural behaviour may not be sufficient to highlight the differences and intensities of emotional states during anticipatory behaviour.

Vocalisations may be an interesting means of exploring the emotional content of the anticipation phase. Indeed, vocalisations have an emotional content in many species^15^. However, until now, vocalisations have rarely been included in anticipatory behaviour ethograms. One exception is rats which have frequency-modulated ultrasonic vocalisations (50 kHz)^16^. In horses, low-pitched vocalisations (i.e., nickers) are proposed by Peters et al.^11^ as expressions of positive anticipation but the authors were not able to score them in their study. In pigs, high-frequency vocalisations are suggested to be a good indicator of their emotional state during the anticipation of different events^12^. Pigs are more likely to make high-frequency vocalisations before negative rather than positive events^12^. Pigs are good candidates to study vocalisations during anticipation because the variability in their vocal expressions corresponding to emotions has already been reported on, both according to their emotional valence and the degree of arousal^17–20^. Thus, the quality of vocalisations could be a good indicator to evaluate anticipation in piglets. This might be even more relevant with the quality of grunts, which are expressed in negative and positive situations, and show a large variability in their acoustic properties^17,35^.

In the present study, we wanted to measure the vocal expression in piglets during the anticipation of pseudo-social events having possibly positive values. In the farming context, pigs may experience various kinds of pseudo-social events. Pigs are social animals and the presence of familiar conspecifics has a highly positive valence^21^. Pigs also experience interactions with humans and have been shown to develop a positive relationship with them after a period of positively reinforcing interactions, such as brushing and calmly speaking, compared to control animals^22–24^. To our knowledge, no evidence of positive anticipation of human presence and contact has ever been shown in pigs, although it has been shown in captive non-domestic animals^25^.

The aim of this study was to test whether piglets reared in groups could vocally express anticipation of the arrival of social partners and the arrival of a familiar human caregiver (both supposedly of positive valence), and if vocalisations were different according to the social characteristics of the reward. We first conditioned piglets to associate a visual and acoustic stimulus with the arrival of familiar conspecifics and another stimulus with the arrival of a familiar human caregiver. Half of the piglets had previously received additional positive contacts with the human prior to the conditioning, leading to two groups of piglets with different degrees of familiarity toward the human prior to the conditioning phase, during which all piglets received positive contacts with the human. To complete the investigation of emotional values of the anticipated event, we carried out one final test in which we delayed the arrival of the expected partner. Delaying the arrival of the reward would create a discrepancy from their expectations^26^ and thus produce a negative emotional state. This test was used as a negative control of the previous positive anticipation. We measured both their behavioural and vocal activity, as well as the acoustic structure of their grunts, before (i.e., initial phase), during (i.e., anticipatory phase), and after the stimulus (i.e., when the arrival of the reward was delayed). We hypothesized that expecting familiar conspecifics has a positive valence and induces a higher arousal state for all piglets, compared to expecting a familiar human, even taking into account the degree of their familiarity. If vocal signals reflect emotional states, we would expect them to have a different signature when anticipating conspecifics compared to a human. If having received additional contact with the human prior to the test conditioning phase modifies the emotional state of piglets, we would expect a different anticipatory vocal signature between groups that had or had not received additional care.

## Results

The dataset was composed of the last three trials of the conditioning and contained five phases: before the stimulus was broadcasted (initial phase − 1 of trials 10 to 12: 10 to 30 s), while the stimulus was broadcasted (anticipation phase 0 of trials 10 to 12: 20 s), after the signal had stopped and when the entrance of the partner was delayed, for testing the discrepancies from expectations (delayed phases 1, 2, 3 of 12th and last trial: 30 s each). Behavioural and acoustic parameters were used to build scores using multivariate analyses carried out in two steps. A linear discriminant analysis was first computed on a subset of data containing the first two phases of trials (i.e., before phase − 1 and during phase 0 the stimulus). The remaining data set (containing the last three phases of the 12th trial: after the stimulus, when the partner was delayed) was compared using two behavioural scores (LD1bev., LD2bev.) and one acoustic spectral score (LD1ac.). Results are focused on two interactions of the full model describing the experimental design: expression of differential anticipation of partners (partner* phase) and effect of familiarity toward the human partner on differential anticipation of partners (partner* treatment). Results on remaining interactions of the model are available as supplementary material (supplementary figure S3 and Tables S1–S3).

### Piglets behave differently when anticipating the arrival of a familiar human or conspecifics

**Figure 1:**
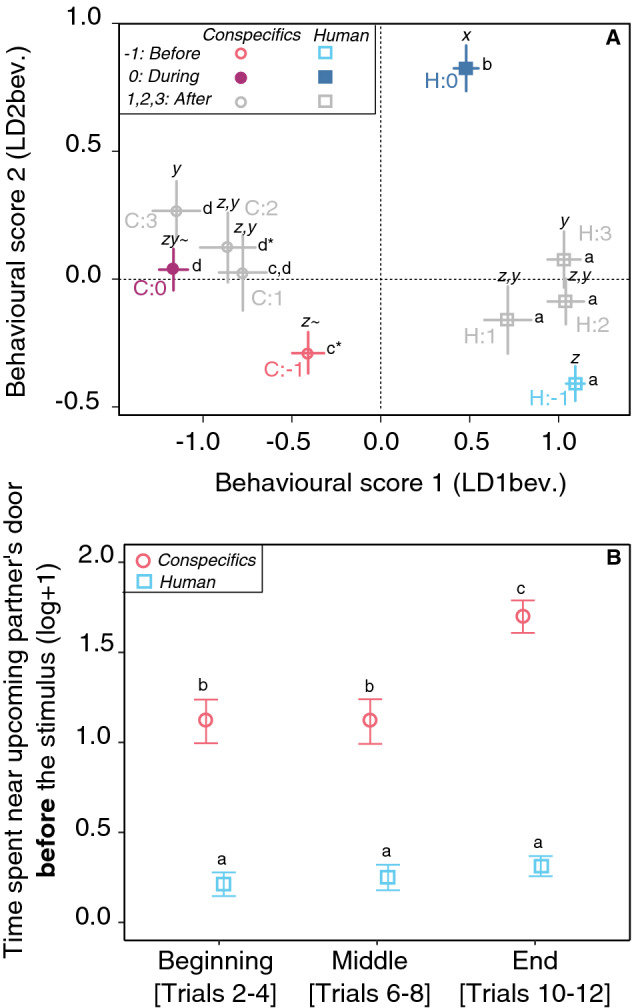
Behavioural responses of piglets during anticipation trials. Trials associated with the entrance of two familiar conspecifics (C) are indicated in grey, red and purple circles, whereas trials associated the entrance of a familiar human (H) are indicated in grey, cyan and blue squares. Circles/Squares and bars represent the mean ± se per group. (**A**) Behavioural space with LD1bev.and LD2bev. scores showing the significant interaction between the phase of the trial (− 1, 0, 1, 2, 3) and the type of partner. Phase -1 corresponds to the time before the broadcasting of a stimulus (initial phase, trials 10 to 12), phase 0 corresponds to the time while the stimulus was broadcasted (anticipatory phase, trials 10 to 12) and phases 1, 2, and 3 are 30 s segments during the phase the piglet was not reunited with a partner as expected (trial 12, ‘discrepancies from expectations’, 90 s in total). Letters from a to d and x to z show significant differences on LD1bev. and LD2bev. respectively. (**B**) Time spent near upcoming partner’s door before the broadcasting of any stimulus (phase-1 only) during the conditioning (grouping trials to create a three sections: beginning, middle and end of the conditioning). Letters shows significant differences between groups. All models use ANOVA tests; estimates and pairwise post hoc tests with Tukey contrasts are available in Tables S1-S3 of the supplementary material.

The first behavioural score (LD1bev.) was negatively correlated with the time spent near the upcoming partner’s door and the time spent watching this door and positively correlated with the number of zones explored (Table 1). Statistics showed a significant interaction between the phase of the trial and the partner (X^2^_4_ = 13.9, *p* = 0.008, Fig. 1A, significance letter from a to d). During trials of anticipation of the human partner, the stimulus led to a significant decrease of LD1bev. compared to the initial phase (H partner, phase − 1 vs. 0, T.ratio = 5.97, *p* < 0.001). After the stimulus, while the arrival of the partner was delayed, LD1bev. increased and then remained stable (H partner, phase 0 vs. 1: T.ratio =  − 1.64, *p* = 0.83, 0 vs*.* 2: T.ratio =  − 3.88, *p* = 0.004, 0 vs. 3: T.ratio =  − 3.82, *p* = 0.005); remaining at the same level as before the stimulus (H partner, phase − 1 vs. 1:2:3, |T.ratio|< 2.62, *p* > 0.21). During trials of anticipation of conspecifics, the stimulus led to a significant decrease of LD1 compared to the initial phase (C partner, phase − 1 vs. 0, T.ratio = 7.33, *p* < 0.001). After the stimulus, LD1bev. did not change while the arrival of the partner was delayed (C partner, phase 0 vs*.* 1:2:3, |T.ratio|< 2.7, *p* > 0.18), but tended to be different than during the initial phase (C partner, phase − 1 vs. 1, T.ratio = 2.5, *p* = 0.25; phase − 1 vs. 2, T.ratio = 3.1, *p* = 0.06; phase − 1 vs. 3, T.ratio = 5.1, *p* < 0.001). Prior to any stimulus, LD1bev. differed depending on the type of partner (phase − 1, C vs. H, T.ratio =  − 14.74, *p* < 0.001).

**Table 1:** Weightings of the behavioural variables on Linear Discriminant Functions (respectively LD1bev. and LD2bev.) of piglets trained to expect the arrival of familiar conspecifics of a familiar human. Rows indicate the parameters used to build the functions and columns indicate their respective weightings on the first two functions.

Behavioural scores	LD1bev	LD2bev
Total time spent freezing	0.062	− 0.181
Averaged duration spent per zone	− 0.044	0.438
Number of zones explored	0.226	0.134
Total time spent watching upcoming partner's door	− 0.224	0.356
Number of times turned toward upcoming partner's door	− 0.188	0.664
Total time spent in zones near upcoming partner's door	− 1.083	− 0.708

Note that this table concerns the data from phase ‘− 1’ (before the stimulus) and phase ‘0’ (during the stimulus) and that a factor taking into account the partner, the phase and the treatment was used to discriminate the groups. This weighting was then used to predict the data gathered during the discrepancy from expectations test (trial 12) and statistics were run on LD1bev. and LD2bev. after projection, as two behavioural scores.

The second behavioural score (LD2bev.) was negatively correlated with the amount of time spent near the upcoming partner’s door and the amount of time spent freezing, and positively correlated with the amount of time spent watching the upcoming partner’s door, the number of times watching the upcoming partner’s door and the total time spent per zone (Table 1). Statistical analysis showed a significant interaction between the phase of the trial and the partner (X^2^_4_ = 49.1, *p* < 0.001, Fig. 1A, significance letter from x to z). During trials of anticipation of the human partner, the stimulus led to a significant increase of LD2bev. as compared to the initial phase (H partner, phase − 1 vs. 0, T.ratio = 11.29, *p* < 0.001). After the stimulus, LD2bev. significantly decreased when the arrival of the partner was delayed (phase 1) and then remained stable (H partner, phase 0 vs*.* 1:2:3 |T.ratio|< 6.37, *p* < 0.001), at the same level as before the stimulus (H partner, phase − 1 vs*.* 1:2:3, |T.ratio|< 3.20, *p* > 0.05). Such effects were not significant for the anticipation of conspecifics, for which the only trend was an increase of LD2bev. during the anticipation phase (C partner, phase − 1 vs. 0, T.ratio =  − 3.01, *p* = 0.08). No difference was found between the anticipation phase and the phase when the arrival of the partner was delayed (C partner, phase 0 vs. 1:2:3 |T.ratio|< 1.48, *p* > 0.90). Before the stimulus, we found no effect of the upcoming partner on LD2bev (phase − 1, C vs*.* H, T.ratio = 1.10, *p* = 0.98).

For both behavioural scores (LD1bev. and LD2bev), there was no evidence of an effect of the treatment depending on the type of partner (partner: treatment, X^2^_4_ < 2.00, *p* > 0.16) and no main effect of the treatment (X^2^_1_ < 0.26, *p* > 0.62) (see supplementary material Table S1).

LD1bev. and LD2bev. were mainly explained by the location of the piglet in the experimental room, and piglets significantly spent more time near the conspecifics’ door (LD1bev., Table 1). Thus, differences between LD1bev. prior to the stimulus could be explained by either location biases in the test room or the expression of a preference toward the conspecific door. To explore this further, we looked at the effect of the trial number (i.e., beginning vs. the middle vs. the end of the experiment, Fig. 1B) on the time spent near the upcoming partner’s door. If piglets expressed a preference toward the conspecific door during the conditioning, the interaction between the trial number and the partner should be significant. Analysis shows a significant interaction between the partner and the conditioning trial number (X^2^_2_ = 11.96, *p* = 0.003); although piglets spent more time near the conspecifics’ door than near the human door, independently from the upcoming partner, piglets increased their time near the conspecifics’ door at the end of the conditioning (C partner, middle vs. end of the conditioning, T.ratio = 4.19, *p* = 0.001), but did not increase their time spent near the human door (H partner, pairwise tests between all trial factors, |T.ratio|< 0.8, *p* > 0.97).

### Piglets grunt at a higher rate when anticipating conspecifics

**Figure 2:**
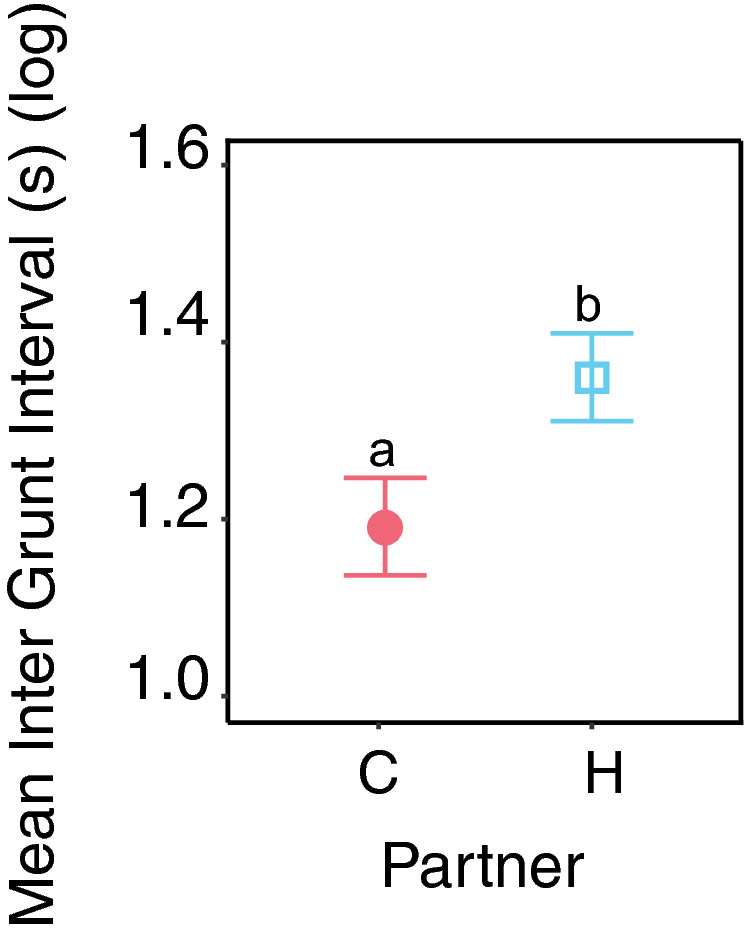
Vocal dynamics in piglets during phase 0 (anticipatory phase). Mean ± se inter-grunt interval per individual per type of partner. Different letters represent significant differences between expected partners. All models use ANOVA tests; estimates and pairwise post hoc tests with Tukey contrasts are available in supplementary tables S1, S2 and S3.

The rhythm of grunting, while the stimulus was broadcasted (anticipation phase ‘0’ with a fixed duration of 20 s), was tested using the mean individual inter-grunt interval. The inter-grunt interval was significantly lower during anticipation of conspecifics than during anticipation of a human (X^2^_1_ = 4.35, *p* = 0.037, Fig. 2), independently from the treatment (X^2^_1_ = 0.037, *p* = 0.85, table S1).

### The structure of piglets’ grunts differs when anticipating familiar conspecifics versus a familiar human

The acoustic structure of 2270 grunts (see Table S4 for data composition) was analysed using the duration of the call (log(duration)) and a spectral score, i.e., the first linear discriminant function built from nine acoustic parameters representative of the call spectrum (LD1ac., Table 2): a higher score indicates increased spectral noise, whereas a lower core indicates increased pitch content).

**Table 2:** Weightings of the first linear discriminant function (LD1ac.) following the spectral analysis of grunts in piglets.

Parameters	Loadings of LD1ac.
Mean	− 1.442
Median	− 0.161
Mode	0.198
Q25	0.034
Q75	0.308
Centroid	− 1.442
SH	2.888
SFM	2.249
Entropy (H)	− 2.817

Rows indicate the parameters used to build the functions and columns indicates their respective loadings on the first function. The linear discriminant analysis was made with the data before and during the stimulus (phases − 1 and 0). A factor taking into account the partner, the phase and the treatment was used to discriminate or not the groups.

**Figure 3:**
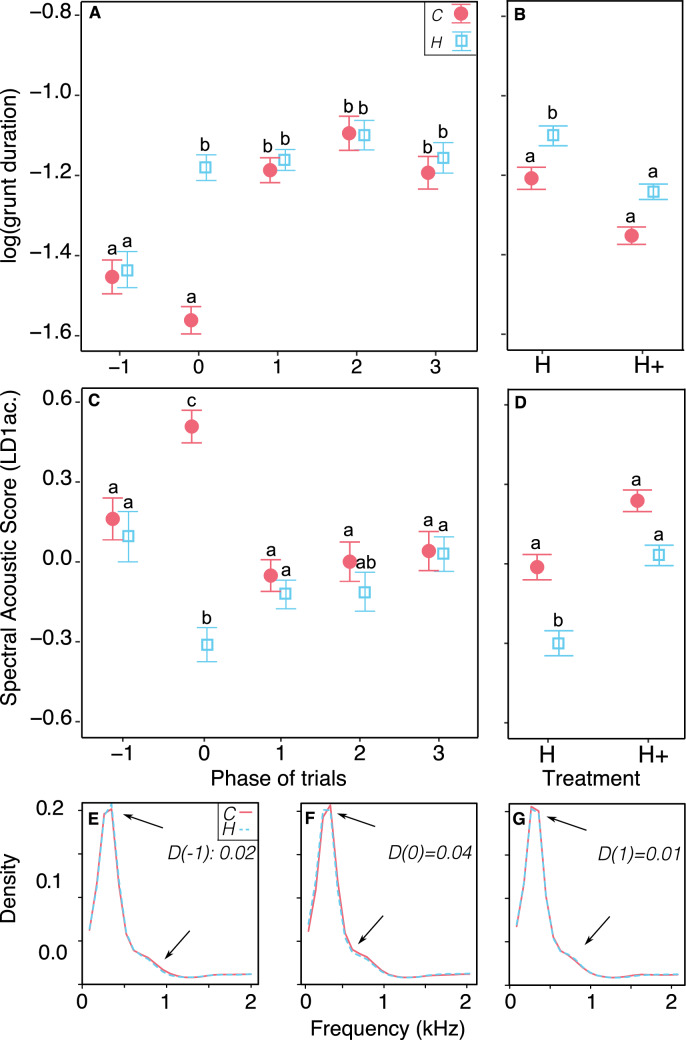
Acoustic structure (mean ± se) of piglets’ grunts depending on the type of partner: familiar conspecifics (C, filled red circles) or human (H, empty cyan squares), and treatment (H + , additional contacts group or minimal contact group (H). (**A**) and (**C**) changes in grunt duration (**A**) and spectral acoustic score (**C**) during different phases of anticipation trials, independently from the treatment. Phases correspond to: before the stimulus (phase − 1), during the stimulus i.e., anticipation phase (phase 0) and after the stimulus, i.e., during phases of discrepancy from expectation (phases 1, 2, 3, each of 30 s). Grunt duration (**B**) and spectral acoustic score (**D**) according to the type of partner and treatment, independently from the phase of the trial. Letters show significant differences between groups. All models use ANOVA tests; estimates and pairwise post hoc tests with Tukey contrasts are available in tables S1, S2 and S3 respectively in supplementary material file. (**E**–**G**): comparison of mean spectra between types of partner per phase in the main frequency range of the grunts (0.2–2 kHz), for which the coefficient D(phase) corresponds to a metric of spectral dissimilarity (0 < D < 1, computed with ‘diffspec’ function, ‘seewave’ R package). Arrows indicate where the changes are the strongest. Within the entire 0.2–8 kHz frequency range studied, the third quartile (Q75) of all grunts had a mean of 2157(± 72) Hz (supplementary Table S4), so only the range 0.2–2 kHz is illustrated here. Due to extremely low variability in the spectrum per group, standard errors of the mean of all spectra are not visible on the plots. The number of grunts used per group is available in Supplementary Table S4 (56 < N < 241, median = 101 grunts on a total of 2270).

The social quality of the partner (conspecifics vs. familiar human) had an effect along phases of trials in regards to both the duration of grunts and their spectral structure (phase: partner interaction, X^2^_4_ = 50.3, *p* < 0.001, Fig. 3A and X^2^_4_ = 63.5, *p* < 0.001 Fig. 3C respectively).

The duration of the grunts was longer during the anticipatory phase of the human partner than before the stimulus (partner H, phase − 1 vs. 0, T.ratio =  − 4.79, *p* < 0.001) and remained longer after the stimulus when the arrival of the human was delayed (partner H, phase 0 vs. 1:2:3, |T.ratio|< 1.66, *p* > 0.82, phase − 1 vs. 1:2:3, |T.ratio|> 3.59, *p* < 0.012). Contrary to human trials, in conspecifics trials, the stimulus did not affect grunt duration (partner C, phase − 1 vs*.* 0, T.ratio = 2.42, *p* = 0.315). However, grunts became longer after the stimulus when the arrival of conspecifics was delayed (partner C, phase 0 vs. 1:2:3, |T.ratio|> 7.32, *p* < 0.0001). Within phases, the duration of grunts differed between the type of partner only during the anticipation phase (phase 0, C vs*.* H, T.ratio =  − 8.29, *p* < 0.001) but not during other phases (phases − 1:1:2:3, C vs. H, T.ratio < 0.34, *p* = 1.00).

The acoustic spectral score LD1ac. (Table 2) decreased during the anticipation phase compared to the initial phase of trials of anticipation of the human partner (H partner, phase − 1 vs*.* 0, T.ratio = 3.95, *p* = 0.003) and increased after the stimulus when the human was delayed (H partner, phase 0 vs. 1:3, |T.ratio|> 3.74, *p* < 0.007, phase 0 vs. 2*:* T.ratio =  − 2.85, *p* = 0.12), returning to LD1ac. values measured before the stimulus (H partner, phase − 1 vs. 1:2:3, |T.ratio|< 1.29, *p* > 0.96). For trials of anticipation of conspecifics, LD1ac. increased during the anticipation phase compared to the initial phase (H partner, phase − 1 vs. 0, T.ratio =  − 3.97, *p* = 0.003) and decreased after the stimulus when the arrival of conspecifics was delayed (C partner, phase 0 vs. 1:2:3, |T.ratio|> 4.90, *p* < 0.001), returning to LD1ac. values measured before the stimulus (C partner, phase − 1 vs. 1:2:3,|T.ratio|< 0.66, *p* = 1.00). Within phases, LD1ac. was significantly different between partners only during the anticipation phase (phase 0, C vs. H, T.ratio = 9.00, *p* < 0.001), but not within any other phases (phases − 1:1:2:3, C vs. H, |T.ratio|< 0.66, *p* = 1.00).

To illustrate spectral changes in grunts, comparisons of mean spectra between types of partner per phase are shown in Figs. 3E–G within the main frequency range of the grunts (0.2–2 kHz).

### Familiarity with the human affects the acoustic structure of grunts in response to the human or to conspecific partners

A significant interaction between the familiarity with the human and the type of partner anticipated was found for both acoustic descriptors of grunt structure (treatment: partner interaction, X^2^_1_ = 5.84, *p* = 0.016, Fig. 3B, and X^2^_1_ = 6.45, *p* = 0.010, Fig. 3D respectively for grunt duration and LD1ac.). When anticipating the human, during all phases, piglets from the H group (low familiarity) produced longer grunts than piglets from the H + group (higher familiarity) (H partner, H vs. H + , T.ratio = 2.69, *p* = 0.04) but there was no difference during trials of anticipation of conspecifics (C partner, H vs. H+ , T.ratio = 1.31, *p* = 0.56, Fig. 3B). Concerning the acoustic spectral score (LD1ac.), when anticipating the human, during all phases, piglets from the H group produced grunts with a lower LD1ac. than when anticipating conspecifics (H group, C vs. H, T.ratio = 4.02, *p* < 0.001). However, no differences in LD1ac. between partners was found in piglets from the H + group (H + group, C vs. H, T.ratio = 1.37, *p* = 0.52, Fig. 3D).

## Methods

### Ethical note

The study was approved by the ethic committee CREEA and received the authorization no. APAFIS#17071-2018101016045373_V3 from the French Ministry of Higher Education, Research and Innovation; and was in agreement with the French and European legislation regarding experiments on animals.

### Subjects and housing conditions

Sixty weaned female piglets (in two replicates), *Sus scrofa domesticus*, bred from crosses between Large White and Landrace females and Piétrain males were used for this study from 28 to 62 days after birth. Animal housing and experiments took place at the experimental unit UEPR (UE 1421, INRAE France).

One piglet was removed in the middle of the experiment due to health issues independent from the experiment. Piglets from the same litter and having similar weight (< 1 kg difference) were housed by three in a 1.2 × 1.3 m pen on plastic duckboard and panels visually isolated the pens. One bare chain per pen was used for enrichment. Food and water were available ad libitum. Artificial lights were turned on from 8:00 to 17:00 and temperature was maintained between 26 and 27 °C. Two identical rooms were used (5 pens per room per replicate).

### Experimental treatment: human additional contacts: taming period

From day 28 to day 41 of life, two experimental groups were generated as follows:

- *A group with minimal human contact, H group* Control piglets from 10 rearing pens received the minimal amount of daily contact with a stockperson (a 1.70 m tall male who did the feeding, cleaning and health checkups). The stockperson wore a dark green shirt and pants and brown shoes.
- *A group with additional human contact, H + group* in addition to the daily care given by the same stockperson as for H group, piglets from the 10 other rearing pens received sessions of additional human contacts with one of the two experimenters (both women, both between 1.70 and 1.73 m tall, balanced number of pens attributed to each of them). The experimenters wore the same overalls and boots each time they interacted with the piglets: blue overalls and dark green boots. There were twenty-nine sessions of interaction, each 10 min, from day 28 (weaning) until day 39, occurring daily except for weekends. Three sessions per day were performed (except on the day of weaning during which only two were done with a 2-h break in between). Each session took place in the rearing pen and the order of the pen was balanced across days. The handling procedure, using gentle tactile contacts is described in the supplementary material and was similar to Tallet et al.^21^.

At the end of the interaction period, only one group was tamed (H+). We confirmed that the additional human contact treatment induced a positive attraction toward the human in a standard human-piglet reunion test (supplementary material, Fig. S1).

### Two-way associative learning and induction of anticipation: conditioning period

Pen piglets were habituated to the test room for 10 min, two days before the start of the conditioning. The conditioning took place between day 42 and 62 after weaning and lasted twelve days, with two trials per day and at least three hours between trials on the same day.

#### Pseudo-social events

All piglets were individually trained to learn to associate two different stimuli with the arrival of two different (pseudo)-social partners followed by a 2 min reunion with: either three pen mates (partner = Conspecifics) or a familiar human (partner = Human). When entering the room, the human sat on a bucket and positively interacted with the piglet, in the same manner that additional contacts was provided to the H + group during the taming period (see above section and detailed procedure in supplementary materials). At the beginning of the conditioning phase, piglets from the H + group were already familiar with the human from the taming period, whereas piglets from the H group were unfamiliar with the human and only became familiar during the conditioning. Since additional positive contacts occurred during the conditioning in both experimental groups (H and H+) the human could be considered as familiar for all piglets at the end of the conditioning period, with a different degree of familiarity between the two groups.

#### Associative learning stimuli

**Figure 4:**
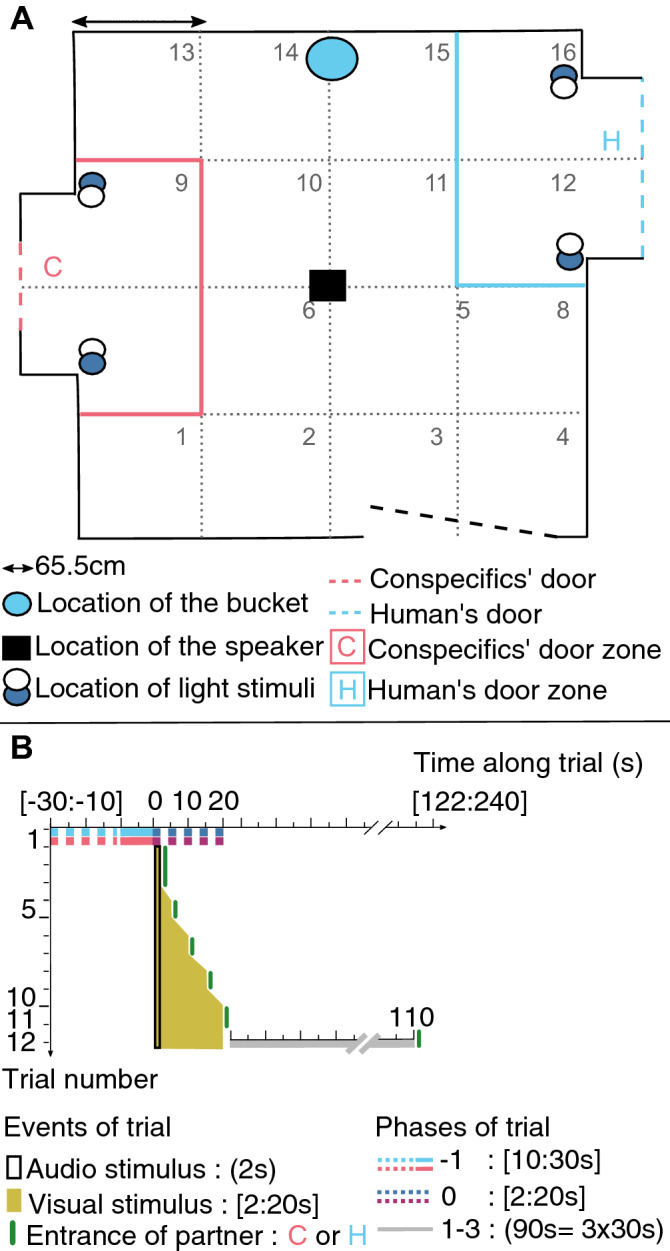
Two-way associative learning: experimental room (**A**) and trial steps (**B**). (**A**) The acoustically isolated room contained three doors: the human’s door (H, in blue on the right), the conspecifics’ door (C, in red on the left), and the entrance door (at the bottom), which remained in place during the entire experiment. A speaker was located in the centre of the room 1 m above the ground and broadcast a 2 s audio stimulus associated with the upcoming partner. Blue or white light around the partner’s door were used as visual stimulus and announced the entrance of the partner. When the human entered the room, they would place a bucket on the floor and sit on it for two minutes, giving additional care to the tested piglet. For behavioural analyses, the room was separated into 16 zones to allow for quantifications of mobility and location in the room as well as the partner door zones (either C or H). (**B**) Steps of each trial. The trial number is indicated on the vertical axis and the time of the trial (in seconds) on the horizontal axis. The ‘0’ indicates the start the audio-visual stimulus. The audio stimulus always lasted for 2 s (empty black area) whereas the duration of the visual stimulus increased during the conditioning, from 2 to 20 s, which is indicated for each trial on the figure (yellow filled area). The green vertical line indicates the entrance of the partner (either C or H). Each trial was divided into several phases: phase ‘ − 1’ corresponds to the initial phase, starting from the entrance of the tested piglet and ending at the start of the stimulus. This phase lasted at least 10 s (solid cyan and red lines) but the duration was randomly set between 10 and 30 s to avoid habituation effects (dotted cyan and red lines). The phase ‘0’ corresponds to the phase of the stimulus onset and lasted from 2 to 20 s depending on the trial (dotted blue and purple lines), and was called the anticipation phase in trials 10 to 12 (used for the analyses). The phases ‘1’, ‘2’, ‘3’ correspond to additional phases of the 12th trial, for which, the entrance of the partner was delayed for 90 s (solid grey line). The 90 s were divided into three segments of 30 s (Phases 1, 2, 3), since the maximum duration of the initial phase was set at 30 s. ‘[]’ indicates interval with variable durations, ‘()’ indicates interval of fixed durations for a given trial.

Associative learning stimuli were chosen to facilitate learning since the aim was not to test learning abilities but the way in which piglets would anticipate the reunions. One stimulus announcing the entrance of a partner combined a visual and an auditory stimulus: lights (blue or white^27^) on a nearby door and auditory stimuli tones (296 Hz or 3100 Hz^28^) broadcasted from a speaker (Mipro MA-100su, Mipro Electronics Co, Taiwan). Four visual-auditory combinations were created and their playback was balanced across all experimental piglets. Sound and light announced the beginning of a new phase of the test and turning off the lights marked the end of a phase and the beginning of the next one. The duration of the audio playback was fixed to 2 s and the duration of the visual part of the stimulus was gradually increased from 2 to 20 s during the conditioning phase (see “Inducing an anticipation phase” section below and Fig. 4B).

#### Associative learning trials

Twenty-four trials were run by piglet; 12 with each partner (Fig. 4B). For each trial, the target piglet entered the experimental room and remained alone for 10 to 30 s before the stimulus started; the duration of this initial was randomized to avoid habituation to the stimulus (phase − 1) which lasted between two and 20 s (phase 0, anticipation). After the end of the stimulus, the partner entered the room. Piglets from the same pen were tested one after the other and the order was randomized from one trial to the next in order to avoid confounding effect of the order within one pen. The order of the pens was randomized from one day to the next in order to avoid confounding effect of the time of day. Piglets were reunited once with each of the possible partners every day (alternating between the morning and the afternoon), except on days 6 and 8 for which they were reunited with the same partner in the morning and in the afternoon in order to avoid habituation to alternating reunions. This design was inspired by Reimert et al.^29^.

#### Inducing an anticipation phase

To generate an anticipatory phase (phase 0) prior to the arrival of a partner, the duration of the stimulus was gradually increased during the conditioning (Fig. 4B)^29^ [trials 1–3: two seconds, trials 4–5: five seconds, trials 6–7: 10 s, trials 8–9: 15 s, trials 10–12: 20 s]. To allow the recording of the vocalisations produced during the anticipatory phase, only the visual stimulus was prolonged, whereas the auditory stimulus was kept at two seconds for all trials. Only trials 10 to 12, containing a stimulus of 20 s, were analysed.

#### Testing anticipation: test of discrepancies from expectation

In order to test for positive anticipation, we chose to change the associative learning paradigm with a negative control on the 12th and last trial. To do so, we delayed the entrance of the positive expected partner: the stimulus stopped but the partner entered only after 90 s (Fig. 4B). Thus, the piglet remained alone (known as a negative situation) instead of being immediately reunited with its pen mates or the familiar human (positively rewarding situations). The delay of 90 s was long enough to make the arrival unpredictable and mimic a long absence of the expected reward, considering the duration of a normal trial. If piglets learned the two-way associative paradigm, this last test would lead to a discrepancy from their expectation and allow us to track changes in emotional states^26^. In order to study each phase using a similar timing and to study the changes in activity, we divided the delayed period into three 30 s phases (named phases 1, 2 and 3). We choose 30 s as it was the maximum duration of the randomly set initial phase (Phase − 1).

### Behavioural measures

Behaviours were monitored using a camera (Bosh, Box 960H-CDD) and annotated using *The Observer XT 14.0* (Noldus, The Netherlands) software. The square room was split into 16 equally-dimensioned zones to assess the mobility and exploratory behaviour of the piglet. The following behaviours were monitored and standardised per minute for each phase: the time spent near conspecifics’ and human door zones, time spent watching conspecific and human doors, the number of times the piglet watched the conspecifics’ and human’ doors, the number of zones explored, the average time spent per zone, and the time spent not moving in the room. A zone was considered crossed when the anterior legs of the piglet crossed into a virtual zone (Fig. 4A). The watching behaviour was quantified as when the piglet turned their head toward the door in question. Behavioural scores were then calculated to quantify global responses (see below).

### Acoustic measures and analyses

#### Acoustic monitoring

Vocalisations were recorded with an AKG C314 microphone placed in the center of the room and one meter above the ground, connected to a Marantz MD661MK2 recorder. Vocalisations produced during each phase of the trial were manually annotated according to vocal type (grunt, squeak, bark, scream and mixed calls), after visual inspection of spectrograms on Praat software. Only grunts were analysed further as they were the most frequently expressed. However, additional observational data on other call types are available in the supplementary data (Fig. S2).

#### Acoustic measures of grunts

A spectro-temporal analysis was performed with custom-written codes using the Seewave R package^30^ implemented in R^31^. We first studied the spectral properties of the remaining background noise of the experimental room (electric noises and remaining low frequency noises from the rest of the building), using 20 examples of 0.5 s fragments. Since the first quartile (Q25) of the normalized spectrum of the background noise was 250 Hz and the grunts are low frequency vocalisations, we decided to remove all frequencies below 200 Hz in order to focus on the most relevant frequencies, using a 0.2–8 kHz bandpass filtering (‘fir’ function). As a consequence, all results presented in this study are on a 0.2–8 kHz frequency range, and no conclusions on possible frequency components of grunts below this 200 Hz threshold can be drawn here. A standardised grunt was detected when the amplitude crossed a 5% amplitude threshold (‘timer’ function) to measure the duration. After amplitude normalisation, the following spectral parameters were calculated using the ‘specprop’ function (FFT with Hamming window, window length = 512, overlap = 50%): mean, median, first (Q25) and third (Q75) quartiles, interquartile range (IQR), centroid and mode (all in Hz). The grunt dominant frequency (in kHz) was also calculated (‘dfreq’, 50% overlapping FFTs, window length = 512), which is the mean over the grunt duration of the frequencies with the highest level of energy. Parameters measuring noisiness and entropy of the grunts were: Shannon entropy (sh), Spectral Flatness (Wiener entropy, sfm) and Entropy (H) [combining both Shannon and Temporal envelop entropy, length = 512, Hilbert envelop). Two linear acoustic parameters were used: the logarithm of grunt duration and a built-in spectral acoustic score with all spectral parameters (see below). Table of acoustic data available in supplementary material (table S4).

### Statistical analyses

#### Behavioural and acoustic scores

To assess changes in global behavioural and acoustic responses during the anticipation phase, parameters were used to build scores using multivariate analyses carried out in two steps. First, a linear discriminant analysis was computed on a subset of data containing the first two phases of the test, maximizing differences between groups of an ad hoc factor ‘phase*treatment*partner’. Two behavioural scores (LD1bev. and LD2bev.) and one spectral acoustic score (LD1ac.) were built. On the remaining dataset (trial 12: phases 1, 2, 3 for which the entrance of the partner was delayed), a projection was computed using LDs scores, allowing analysis of the differences in behavioral/acoustic space(s).

#### Statistical tests and validation

We tested for differences in LDs scores since the purpose of using a delayed entrance of the partner was to know whether the piglets would keep the same state that they had during anticipation, return to the state they had in the initial phase or exhibit an intermediate response. All statistics were carried out on R^31^. A linear mixed effect model (‘lmer’ function, ‘lme4’ R package) was built to test two-way interactions between the different factors ‘phase of the trial’ (phases: − 1, 0, 1, 2, 3), ‘partner’ (Human or Conspecifics) and ‘treatment’ (additional H + or minimal human contacts H). The factor ‘replicate’ (first or second) was also tested in interaction with ‘treatment’ and ‘partner’. Piglet identity was used as random factor to take into account repeated measures. This model was used to test for behavioural scores (LD1bev. and LD2bev.), the spectral acoustic score (LDac.) and the duration score (log). For vocal rhythm (inter-grunt interval), the model was simplified to only study of the anticipation phase (phase 0), since the metric highly depended on the number of observations. The following two-way interactions were tested: ‘partner’ and ‘treatment’, ‘replicate’ and ‘partner’, ‘replicate’ and ‘treatment’. To test for biases in the piglet’s location in the room prior to the playback of any stimulus (phase − 1), the time spent near to the upcoming partner’s door (parameter loading the most on the LDs), was used as response variable and trials were grouped in a three-level factor: ‘beginning: trials 2–4’, ‘middle: trials 6–8’ and ‘end: trials 10–12’. The model tested the three-way interaction between ‘trial’, ‘partner’ and ‘treatment’ and two-way interactions between ‘replicate’ and ‘partner’ or ‘treatment’. All linear models were validated by visual inspection of the symmetrical and normal distribution of the residuals (‘plotresid’ in ‘RVAideMemoire’ R package^32^). Anovas were computed on models to test for significant effects of explanatory variables (‘car’ R package^33^). Model estimates and pairwise post hoc tests were computed using Tukey correction for multiple testing (‘lsmeans’ R package^34^). A complete report of statistics is available as supplementary material (tables S1-S3).

## Discussion

This study was aimed at analysing the acoustic expression of anticipation of pseudo-social events: the arrival of two pen mates or of a familiar human, followed by putatively positive reunion time with the pen mates or with a human (providing additional positive human contact to the piglet). After introducing an anticipation phase, the occurrence and positive aspect of it was tested by delaying the entrance of the partner for a relatively long time in proportion to the normal duration of the trial, creating a discrepancy from piglets’ expectations and inducing a negative state. If piglets are able to positively anticipate the entrance of a pseudo-social partner, then their emotional state should change during this later phase. If they differently anticipate the reunion with their pen mates versus the reunion with a familiar human, this should be reflected in the expression of their anticipation.

The behavioural analysis showed that piglets express the anticipation of the pseudo-social reunions by a short-term specific response during the anticipation phase compared to the other phases (i.e., approaching the zone where the partners entered during the stimulus phase, attentive behaviours toward this location, and partner-specific vocal expressions). When the arrival of the partner was delayed, the duration of the piglets’ grunts increased for both partner types. Longer grunts had already been associated with a negative emotional valence^19,20,35^, which confirms that delaying the arrival of the partner led to a negative emotional state in comparison to the initial phase: a discrepancy from expectation that produced something like frustration. These results allow us to conclude that we did succeed in generating a specific anticipatory state during the tests which was expressed both behaviourally and vocally. We thus confirm the cognitive ability of weaned piglets for associative learning, and for developing expectations from their environment^12^.

Piglets did show a preference for their conspecifics compared to the familiar human. Indeed, piglets spent more time near the area of the room where the conspecifics were supposed to enter during the conditioning sessions prior to the playback of a stimulus, in comparison with the area where the human partner was supposed to enter. In addition, during the delay phase, when expecting conspecifics, piglets expressed reactions similar to the anticipation phase, whereas when expecting a human, they rather showed reactions similar to the initial phase before the stimulus playback. This behavioural data confirm the preference for their conspecifics that represent a stronger positive valence than the arrival of a familiar human. Vocal dynamics differed between partners and was in line with behavioural observations. The inter-grunt interval was lower when piglets were expecting conspecifics. Morton’s rules explain that the rhythm of a behaviour can be positively linked to motivation^36^. Thus, an increase in vocal activity when expecting conspecifics may be explained by the expression of a higher motivation toward this reward compared to the human reward, and thus a higher state of arousal.

This allowed us to measure the vocal expression of anticipation according to the event that was anticipated: the arrival of pen mates or of a familiar human (regardless of degrees of familiarity). In regards to the temporal features of the grunts, we observed longer grunts when anticipating a human. So piglets may express a frustration state already when anticipating a human instead of conspecifics. However, we failed to observe a change in grunt duration during the anticipation of pen mates; as their grunts remained as short as during the initial phase. Two non-exclusive hypotheses can be raised to explain this result: (1) piglets learned that something positive was going to happen when entering the room (either the arrival of its pen mates or the arrival of a human with positive contacts, but not remaining in isolation) and began by expressing a non-specific positive state during the initial phase, so that grunts produced during the initial phase were already ‘non-specific positive grunts’, and (2) the anatomical constraints of piglets’ vocal tract does not allow them to shorten the grunts.

In a recent study, Briefer et al.^19^ showed that vocalisations (but not specifically grunts although they are usually over represented in datasets) recorded in positive contexts lasted 0.34 < 0.42 < 0.51 s. If we look at all of the grunts produced during the initial phase in our study, the average grunt duration is 0.13 < 0.27 < 0.41 s (Table S4). This may be in line with the first hypothesis, although the second hypothesis cannot be ruled out. To disentangle these hypotheses, we would need to measure grunt duration in a two-way associative learning with a positive and a negative social context (e.g., isolation vs. arrival of conspecifics). However, the central aim of the present study was to compare different positive contexts, and the piglets’ grunts during the initial phase should not be biased and would reflect a neutral emotional state.

Piglets expecting a human already produced longer grunts during the anticipation phase in comparison to the initial phase, and the grunts remained long during the delayed phases when the entrance of the human was delayed, similarly to when the entrance of pen mates was delayed. An increase in grunt duration during the anticipation phase may mean that having a human as reward instead of a conspecific may be negatively anticipated. The piglets expressed a similar frustration state during the anticipation phase as when the entrance of any (pseudo)social partner was delayed. We found an average duration for the phase of (0.31 < 0.35 < 0.39, table S4), value that are similar to what Briefer et al.^19^ found for negative contexts. This result is surprising because our behavioural data show that additional contact has a positive effect on the human-piglet relationship (Fig. S1). Thus, for at least half of the piglets, we can conclude that the presence of the human was positive as compared to being isolated before the conditioning took place. Moreover, the additional human contact treatment had no effect on behavioural data (Table S1) or on grunt duration when comparing the initial and anticipation phases (Fig. S3, table S3). Thus, we can conclude that both reunions were positive and that an increase in the degree of familiarity towards the human arises during the conditioning. Since anticipation amplifies emotional states^4,37^ and because during the conditioning there were two different positive outcomes that may arise in one trial (either the arrival of a familiar human or conspecifics), we can hypothesize that piglets experience a cognitive bias. Piglets may rank the two possible outputs, increasing the positivity of the arrival of conspecifics and increasing the negativity of having a human instead of a conspecific. In that case, the vocal expression of anticipating a human may already be the expression of a frustration rather than a positive emotion of lower intensity as compared to anticipating conspecifics.

Spectral features of grunt changed drastically regarding the quality of the partner and contrary to grunt duration, the changes were specific to the anticipation phase. Indeed, the spectral score increased when anticipating pen mates and decreased when anticipating a human, but returned to values similar to those observed during the initial phase after the stimulus, when the entrance of the partner was delayed. Therefore, the acoustic spectral score did not vary the same way depending on the quality of the partner: when expecting conspecifics, piglets produced grunt with higher spectral noise, whereas they produced grunts with a higher frequency range and greater temporal noise when expecting a human. Since arousal has been linked to changes in the environment in other mammals (rodents^38^), we can hypothesize that these rapid spectral changes are linked to rapid changes in the emotional arousal of piglets. Indeed, harmonicity decreases with arousal in grunts^17^. Another physiological measure would confirm such a hypothesis: for example, heart-rate and its variability would be good indicators the arousal of the animals during the anticipation phase, independent of valence^12^.

Regarding the effect of the taming treatment, we found no evidence of an effect of the treatment on behavioural activity, but the spectro-temporal features of grunts were different in non-tamed piglets when those trials were associated with a human partner. Since prior to the conditioning, half of the piglets had received additional repeated positive contact sessions with a human which was then prolonged for all piglets during the conditioning, two degrees of familiarity with the human had been generated. We hypothesize that if the degree of familiarity with the human impacts the way piglets behave or vocalise, then we should expect a significative interaction between the social quality of the partner and the taming treatment. Our behavioural activity results differ from the prediction, but the spectro-temporal features of the grunts are in line with it: non tamed piglets produce longer, less noisy and higher pitched grunts when trials are associated with a human, whereas no difference is significant in tamed piglets. Thus, we can hypothesize that having a lower degree of familiarity changes the vocal expression specifically toward the human and may lead to higher reactivity. Less familiarised piglets would be more reactive to negative contexts related to human presence than more familiarised piglets. In any case, we demonstrate that analysing the vocal structure of grunts tells us more information about the piglets’ emotional states than using only behavioural monitoring. It would be interesting to add a physiological measure to confirm the results (e.g., heart rate, as in Baciadonna et al. 2020^13^).

To conclude, we showed that piglets were able to express behavioural and vocal flexibility when anticipating pseudo-social events. In addition, grunts were spectrally and temporally different whether they were expecting a social reunion or an arrival of a familiar human. More interestingly, we also showed that analysing spectro-temporal properties of grunts allowed us to distinguish between different contexts (e.g., a discrepancy from expectation, the experience of positive human handling). Thus, acoustic analyses and especially of grunts (which are the most expressed type of vocalisations in pigs), allow for tracking subtle changes in emotional states that behavioural analyses could not. Our new results on the vocal features of grunts during pseudo-social positive contexts illustrate the possibility of a better exploration of emotional states in non-verbal animals by analysing their vocalisations rather than merely using behavioural investigations.

## Supplementary information

Supplementary Information

## Data Availability

The datasets used and/or analysed during the current study are available at https://doi.org/10.15454/C4JRPP^39^.
