# Supplementary material for: Piglets vocally express the anticipation of pseudo-social contexts in their grunts
Source: Sci Rep. 2020 Oct 28;10:18496. doi: 10.1038/s41598-020-75378-x (PMC7595114; doi:10.1038/s41598-020-75378-x)
Supplement: Supplementary file 1 — Supplementary Information [file 41598_2020_75378_MOESM1_ESM.docx]

Title

Piglets vocally express the anticipation of (pseudo)-social contexts in their grunts

Authors

A.S. Villain^1*^, A. Hazard^1^, M. Danglot^1^, C. Guérin^1^, A. Boissy^2^, C. Tallet^1^º

[1] PEGASE, INRAE, Institut Agro, 35590 Saint Gilles, France

[2] INRAE, Université Clermont Auvergne, Vetagrosup, UMRH, 63122 St-Genès Champanelle, France

Corresponding authors :

[* avelyne.sylvie.villain@gmail.com](mailto:*%20avelyne.sylvie.villain@gmail.com),

º [celine.tallet@inrae.fr](mailto:celine.tallet@inrae.fr)

Supplementary data file

In this file are gathered all supplementary information not required to understand the study but providing more information than the main document and may be useful to replicate the experiment or use it for further meta analyses.

Methods

## additional human contacts procedure

Similarly to Tallet *et al*.^1^, the last phase was adapted to the reaction of each animal and included four steps: (1), the handler hold out the hand towards the animal; (2) if the piglet did not move away, the handler tried to touch it; (3) if the piglet accepted being touched, the handler softly stroked it along the body with the palm of her hand; and (4) once it accepted being stroked, the handler scratched it along the body with her fingers. Scratching consisted in rubbing the skin of the piglets with the finger tips and applying more pressure than stroking. No specific body part of the piglets was more considered that another. Both experimenters trained, prior to the experiment, with non-experimental piglets to standardise the way the handling procedure was held out. In addition, the handler spoke to the piglet with a soft voice from the time she sat on the bucket until the end.

## Human-piglet reunion standard trial before conditioning

To assess the success of the additional human contact treatment in promoting a positive attraction of the piglet toward the human and generating two groups of experimental piglets (H+ vs. H), a standard test of five-minute reunion with the human after five minutes of social isolation^2^ was performed before the conditioning. Since at this stage of the experiment, only the H+ group had received additional human contact, the human was familiar for the H+ group and unfamiliar for the H group.

Results

## additional human contacts increase human-piglet familiarity.

An Anova on the model testing the interaction between the treatment and the replicate, in addition to the experimenter identity showed no interaction between the treatment and the replicate (X^2^_1_ = 0.005, p = 0.94), a main effect of the treatment (X^2^_1_ = 15.2, p = 0.0028) and no effect of the experimenter (X^2^_1_ = 0.51, p = 0.48). Piglets that had received additional human contacts spent more time near the human (< 0.7 meter) (mean ± se, H+: 99.3 ± 12.4, H: 43.4 ± 11.9 seconds) over the five minutes. This trial allowed us to validate that H and H+ groups add different degrees of familiarity with the human (see additional figure S1) and to remove the factor of the experimenter in further analyses. This is a rapid validation of the taming protocol that have already been published before^2^.

**Figure S1: Effect of post weaning additional human contact on piglet’s proximity to a human**. Mean ± se per group (H: control, H+: additional human contact).

## Vocal types produced differs depending on the type of partner

General vocal activity was studied using the number of calls of the different categories pigs produce (grunts, squeals, screams, barks, mixed-calls). First, an analysis of the quality and quantity of each call types depending on the partner, the phase of trials and the treatment was carried out. Due to non-converging models (explained by the unbalanced occurrence of call types and the number of individuals contributing to them), no reliable statistics could be run on the occurrence of call types specific to phases, partner or treatment. However, it has to be noticed that some call types only occurred in some groups: for instance, screams or squeals were exclusively associated to the human partner, bark were associated to the anticipation phase (phase 0, figure S2). This is the reason why, only grunts were considered in the study, since they were present in all groups and produced by a high enough number of individuals in each of the groups. This reflects the recent studies on vocal expression of identity or of emotions focusing on grunts^3-5^. Considering the number of grunt produced per phase/treatment/partner, statistics showed no significant interactions between the treatment, the partner and the phase of trials on the number of grunts produced but a significant effect of the phase (see table S1, S3). No effect of the partner nor treatment were found (X^2^_4_=2.78 p=0.10, X^2^_4_=0.180 p=0.67 respectively).

**Figure S2: General vocal activity during the anticipation trial according to the phase of the trial** (-1, 0, 1, 2 and 3)**, the type of partner** (C: conspecifics, H: human) **and the treatment** (H+: additional contacts, H: minimal contact). Phase -1 corresponds to the time before the broadcasting of a signal, phase 0 corresponds to the time during the signal and phases 1, 2, and 3 are 30 second segments during the violation of expectation period (90 seconds in total). Vocal activity was computed according to the number of calls of each call type produced per group and the rhythm of production. Mosaic plot representing the relative proportion of each call type (grunt, squeal, scream, mixed, bark) according to the partner, the treatment and the phase. The number within each box represents the number of individuals contributing to the counting. When less than three individuals contributed, the identity of the piglet is also written in the box (a to i letters) to allow an illustration of repeated individuals for several categories. The box size represents the relative number of calls, and a scale of the size for 10 calls is illustrated. No statistics could be run to analyse the quality and quantity of calls types depending the partner and treatment due to non-converging models.

## Familiarity toward the human slightly affects the structure of anticipatory grunts

In the model, reflecting the experimental design, the effect of the familiarity toward the human partner along phases of trials was included and was significant for both grunt duration and spectral acoustic score (LD1ac.) (phase: treatment interaction, X^2^_4_ = 12.4, p = 0.015 figure S3A, and X^2^_4_ = 14.8, p = 0.005 figure S3B respectively).

Regarding grunt duration, in the H+ group (handled piglets before the conditioning), we found no difference in the duration of grunts during the anticipation phase compared to the other (H+ group phase 0 *vs.* -1:1:2:3, |T.ratio| < 2.95, p > 0.094) but grunts were longer after the stimulus, and when the arrival of the partner was delayed, than before the stimulus (H+ group phase -1 *vs.* 1:2:3, |T.ratio| > 3.75, p < 0.007). In the H group, the duration of grunts increased after the stimulus, and when the arrival of the partner was delayed (group H, phase 0 vs. 1:2:3, |T.ratio| > 3.38, p < 0.025) but did not differ between the anticipation phase and the initial phase (group H, phase -1 vs. 0, T.ratio = 0.23, p = 0.55).

Regarding the spectral score LD1ac., in the H+ group, a significant decrease of LD1ac. was found from the phase before the stimulus to the first phase while the arrival of the partner was delayed (H+ group, phase -1 *vs.* 1, T.ratio = 3.26 p = 0.038) and all other comparisons between phases did not differ (H+ group, all other phases, |T.ratio| < 2.89, p > 0.11). In the H group, no difference was found in LD1ac. between all phases (H group, pairwise between all phases |T.ratio| < 1.80, p > 0.73), Within phases, no difference was found between treatments but LD1ac.in the H+ group tended to be higher than LD1ac.in the H group before the stimulus was broadcasted (phase -1, H vs. H+, T.ratio = -3.06, p = 0.078). All other comparisons between treatments were non-significant (all phases, H vs. H+, |T.ratio| < 2.69, p > 0.19).


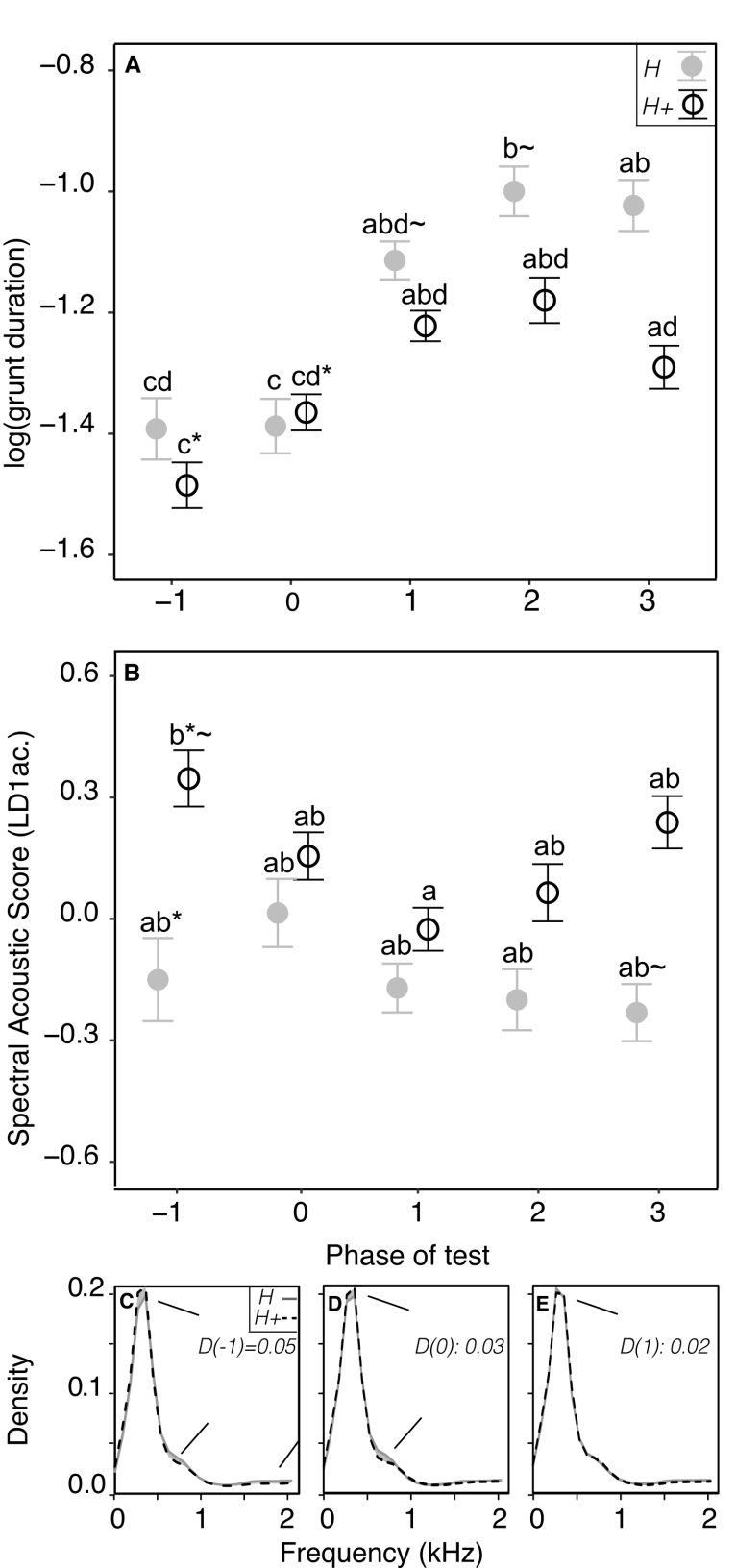


**Figure S3:** Acoustic structure (mean ± se) of piglets’ grunts depending treatment [additional contacts group (H+, black empty circles) or minimal contact group (H, grey filled cicles) and the phase of trials. A and C : evolution of grunt duration (A) and spectral acoustic score (B) along phases of anticipation trials, independently from the partner. Phases correspond to: before the stimulus (phase -1), during the stimulus i.e anticipation phase (phase 0) and after the stimulus, i.e during the violation of expectation phases (phases 1,2,3 of 30 seconds). Letters shows significant differences between groups. All model anova tests, estimates and pairwise post hoc tests with Tukey contrasts are available in tables S1, S2 and S3 respectively as supplementary material. C-E: comparison of mean spectra between types of partner per phase in the main frequency range of changes (0.2-2kHz), for which the coefficient D(phase) correspond to a metric of spectral dissimilarity (0<D<1, computed with ‘diffspec’ function, ‘seewave’ R package). Arrows indicate where the changes are the strongest. Since, overall the studied 0.2-8kHz frequency range, the third quartile (Q75) of all grunt was mean(± se) 2157(± 72) Hz (supplementary table S4), only a zoom in within the 0.2-2kHz was illustrated here. Due to extremely low variability in the spectrum per group, standard errors of the mean of all spectra are not visible on the plots. The number of grunts used per group is available in supplementary table S4 (56<N<241, median=101 grunts on a total of 2270).

## Statistical tables

**Table S1: Anova table for all models computed in the study**. After model validation, effects of explanatory variables were computed using the ‘Anova’ function (‘car’ R package), running Type II Wald chisquare trials. P values are labelled where significant (***: p<0.001, **: p<0.01, *: p<0.05, •:p<0.10). All linear models were computed using the ’lmer’ function, taking into account repeated observations as random factors. Models on counting were computed with a generalized model taking into account repeated observations as random factors, using a Poisson distribution (see model 4).

|  | **Chisq** | **Df** | **P value** |
| --- | --- | --- | --- |
| ***model 1 : Behavioural Score 1 (LD1)*** | | | |
| Partner | 790.937 | 1 | <0.001*** |
| Phase of trial | 90.094 | 4 | <0.001*** |
| Treatment | 0.133 | 1 | 0.715 |
| Batch | 2.706 | 1 | 0.100 |
| Partner: Phase of trial | 13.929 | 4 | 0.008** |
| Partner : Treatment | 1.995 | 1 | 0.158 |
| Phase of trial : treatment | 0.688 | 4 | 0.953 |
| Partner : Batch | 0.035 | 1 | 0.852 |
| Treatment : Batch | 0.030 | 1 | 0.863 |
| Partner : Phase of trial : treatment | 5.232 | 4 | 0.264 |
| ***model 2 : Behavioural Score 2 (LD2)*** | | | |
| Partner | 5.590 | 1 | 0.018* |
| Phase of trial | 105.357 | 4 | <0.001*** |
| Treatment | 0.256 | 1 | 0.613 |
| Batch | 2.556 | 1 | 0.110 |
| Partner: Phase of trial | 49.066 | 4 | <0.001*** |
| Partner : Treatment | 0.069 | 1 | 0.793 |
| Phase of trial : Treatment | 2.594 | 4 | 0.628 |
| Partner : Batch | 0.301 | 1 | 0.583 |
| Treatment : Batch | 0.184 | 1 | 0.668 |
| Partner : Phase of trial : treatment | 1.824 | 4 | 0.768 |
| ***model 3 : log(Total time spent near upcoming partner's door +1)*** | | | |
| Trial (3 levels of factor) | 15.248 | 2 | <0.001*** |
| Partner | 252.790 | 1 | <0.001*** |
| Treatment | 3.789 | 1 | 0.052• |
| Batch | 0.373 | 1 | 0.542 |
| Trial: Partner | 11.956 | 2 | 0.003** |
| Trial: Treatment | 2.799 | 2 | 0.247 |
| Partner: Treatment | 0.000 | 1 | 0.999 |
| Partner: Batch | 0.227 | 1 | 0.634 |
| Treatment: Batch | 4.134 | 1 | 0.042* |
| Trial: Partner: Treatment | 5.208 | 2 | 0.074• |
| ***Model 4: Mean Inter Grunt Interval (log)*** | | | |
| Partner | 4.345 | 1 | 0.037* |
| Treatment | 1.519 | 1 | 0.218 |
| Batch | 0.113 | 1 | 0.737 |
| Partner : Treatment | 0.037 | 1 | 0.847 |
| Partner: Batch | 0.610 | 1 | 0.435 |
| Treatment : Batch | 0.155 | 1 | 0.693 |
| ***model 5 : Spectral acoustic Score (LD1)*** | | | |
| Partner | 22.778 | 1 | <0.001*** |
| Treatment | 3.527 | 1 | 0.060**•** |
| Phase of trial | 4.837 | 4 | 0.304 |
| Batch | 0.033 | 1 | 0.856 |
| Partner : Treatment | 6.453 | 1 | 0.01* |
| Partner: Phase of trial | 63.549 | 4 | <0.001*** |
| Phase of trial : Treatment | 14.762 | 4 | 0.005** |
| Treatment : Batch | 2.305 | 1 | 0.129 |
| Partner : Batch | 12.923 | 1 | <0.001*** |
| Partner : Phase of trial : treatment | 4.207 | 4 | 0.379 |
| ***model 6 : log(Vocalisation duration)*** | | | |
| Partner | 16.434 | 1 | <0.001*** |
| Treatment | 3.964 | 1 | 0.046* |
| Phase of trial | 65.777 | 4 | <0.001*** |
| Batch | 11.948 | 1 | 0.001** |
| Partner : Treatment | 5.843 | 1 | 0.016* |
| Partner: Phase of trial | 50.339 | 4 | <0.001*** |
| Phase of trial : Treatment | 12.414 | 4 | 0.015* |
| Treatment : Batch | 3.841 | 1 | 0.050• |
| Partner : Batch | 4.595 | 1 | 0.032* |
| Partner : Phase of trial : treatment | 6.327 | 4 | 0.176 |

**Table S2: Table of model estimates, following significant effect of explanatory variables**. All estimates were calculated using the ‘lsmeans’ function (‘Lmeans’ R package REF) and are presented per relevant group lsmean, SE, DF, lower.CI upper.CI respectively represent mean estimates, standard error, degrees of freedom and lower and upper limit of 95% confidence interval.

| **Variable 1** | **Variable 2** | **lsmean** | **SE** | **DF** | **Lower.CI** | **Upper.CI** |
| --- | --- | --- | --- | --- | --- | --- |
| ***model 1 : behavioural Score 1 (LD1)*** | | | | | | |
| Partner | Phase of trial | |  |  |  |  |
| C | -1 | -0.411 | 0.080 | 436.097 | -0.568 | -0.255 |
| H | -1 | 1.094 | 0.079 | 433.456 | 0.938 | 1.250 |
| C | 0 | -1.171 | 0.081 | 456.643 | -1.330 | -1.011 |
| H | 0 | 0.477 | 0.081 | 453.933 | 0.318 | 0.637 |
| C | 1 | -0.778 | 0.129 | 940.783 | -1.032 | -0.524 |
| H | 1 | 0.716 | 0.129 | 939.334 | 0.462 | 0.970 |
| C | 2 | -0.859 | 0.129 | 940.783 | -1.113 | -0.605 |
| H | 2 | 1.040 | 0.129 | 939.334 | 0.786 | 1.294 |
| C | 3 | -1.153 | 0.129 | 940.783 | -1.407 | -0.899 |
| H | 3 | 1.032 | 0.129 | 939.334 | 0.778 | 1.286 |
| ***model 2 : behavioural Score 2 (LD2)*** | | | | | | |
| Partner | Phase of trial | |  |  |  |  |
| C | -1 | -0.313 | 0.074 | 716.748 | -0.458 | -0.168 |
| H | -1 | -0.426 | 0.074 | 714.272 | -0.571 | -0.282 |
| C | 0 | -0.002 | 0.076 | 731.417 | -0.150 | 0.147 |
| H | 0 | 0.742 | 0.075 | 729.046 | 0.594 | 0.890 |
| C | 1 | -0.014 | 0.126 | 1016.193 | -0.262 | 0.233 |
| H | 1 | -0.185 | 0.126 | 1015.277 | -0.432 | 0.063 |
| C | 2 | 0.075 | 0.126 | 1016.193 | -0.172 | 0.323 |
| H | 2 | -0.118 | 0.126 | 1015.277 | -0.365 | 0.130 |
| *C* | 3 | 0.214 | 0.126 | 1016.193 | -0.033 | 0.461 |
| H | 3 | 0.036 | 0.126 | 1015.277 | -0.212 | 0.283 |
| ***model 3 : log( Total time spent near upcoming partner's door +1)*** | | | | | |  |
| Trial | Partner |  |  |  |  |  |
| Beginning | C | 1.123 | 0.094 | 204.997 | 0.939 | 1.308 |
| Middle | C | 1.115 | 0.106 | 433.606 | 0.906 | 1.324 |
| End | C | 1.693 | 0.081 | 161.538 | 1.534 | 1.852 |
| Beginning | H | 0.210 | 0.092 | 194.679 | 0.030 | 0.391 |
| Middle | H | 0.243 | 0.106 | 436.376 | 0.034 | 0.453 |
| End | H | 0.309 | 0.081 | 160.583 | 0.151 | 0.468 |
| Treatment | Batch |  |  |  |  |  |
| H | 1 | 0.799 | 0.082 | 152.819 | 0.638 | 0.960 |
| H+ | 1 | 0.814 | 0.078 | 116.846 | 0.659 | 0.968 |
| H | 2 | 0.907 | 0.069 | 106.638 | 0.771 | 1.042 |
| H+ | 2 | 0.618 | 0.070 | 109.605 | 0.479 | 0.757 |
| ***model 4 : Mean Inter Grunt Interval (log)*** | | | |  |  |  |
| Partner |  |  |  |  |  |  |
| C | - | 1.231524626 | 0.061349464 | 66.37954653 | 1.109049541 | 1.353999711 |
| H | - | 1.368648332 | 0.059002793 | 64.47708243 | 1.250858026 | 1.486438637 |
| ***model 6 : Spectral acoustic Score 1 (LD1)*** | | | |  |  |  |
| Partner | Phase of trial | |  |  |  |  |
| C | -1 | 0.102 | 0.104 | 285.030 | -0.101 | 0.306 |
| H | -1 | 0.091 | 0.103 | 287.347 | -0.112 | 0.295 |
| C | 0 | 0.510 | 0.091 | 172.830 | 0.332 | 0.688 |
| H | 0 | -0.322 | 0.091 | 180.161 | -0.502 | -0.142 |
| C | 1 | 0.038 | 0.083 | 124.813 | -0.126 | 0.202 |
| H | 1 | -0.017 | 0.078 | 99.422 | -0.171 | 0.137 |
| C | 2 | 0.045 | 0.093 | 193.451 | -0.138 | 0.229 |
| H | 2 | -0.047 | 0.094 | 198.219 | -0.232 | 0.137 |
| C | 3 | 0.043 | 0.089 | 161.491 | -0.132 | 0.218 |
| H | 3 | 0.127 | 0.091 | 179.062 | -0.052 | 0.305 |
| Partner | Batch |  |  |  |  |  |
| C | 1 | 0.249 | 0.096 | 60.346 | 0.058 | 0.440 |
| H | 1 | -0.092 | 0.094 | 55.780 | -0.280 | 0.095 |
| C | 2 | 0.047 | 0.102 | 66.153 | -0.158 | 0.251 |
| H | 2 | 0.025 | 0.102 | 68.424 | -0.180 | 0.229 |
| Partner | Treatment |  |  |  |  |  |
| C | H | 0.058 | 0.103 | 70.509 | -0.147 | 0.263 |
| H | H | -0.223 | 0.102 | 70.788 | -0.426 | -0.021 |
| C | H+ | 0.237 | 0.096 | 57.934 | 0.046 | 0.429 |
| H | H+ | 0.156 | 0.095 | 56.403 | -0.035 | 0.346 |
| Treatment | Phase of trial | |  |  |  |  |
| H | -1 | -0.166 | 0.127 | 164.945 | -0.417 | 0.085 |
| H+ | -1 | 0.359 | 0.116 | 120.145 | 0.131 | 0.588 |
| H | 0 | 0.037 | 0.118 | 122.512 | -0.195 | 0.270 |
| H+ | 0 | 0.151 | 0.103 | 77.191 | -0.053 | 0.355 |
| H | 1 | -0.045 | 0.104 | 76.524 | -0.251 | 0.161 |
| H+ | 1 | 0.066 | 0.100 | 67.989 | -0.131 | 0.264 |
| H | 2 | -0.118 | 0.115 | 111.674 | -0.344 | 0.108 |
| H+ | 2 | 0.116 | 0.108 | 94.744 | -0.098 | 0.330 |
| H | 3 | -0.120 | 0.112 | 103.783 | -0.341 | 0.100 |
| H+ | 3 | 0.290 | 0.104 | 81.848 | 0.084 | 0.496 |
| ***model 7 : log( Vocalisation duration)*** | | |  |  |  |  |
| Partner | Phase of trial | |  |  |  |  |
| C | -1 | -1.398 | 0.055 | 243.478 | -1.506 | -1.290 |
| H | -1 | -1.390 | 0.055 | 244.706 | -1.497 | -1.282 |
| C | 0 | -1.525 | 0.048 | 152.502 | -1.620 | -1.430 |
| H | 0 | -1.135 | 0.049 | 158.111 | -1.231 | -1.039 |
| C | 1 | -1.215 | 0.045 | 113.647 | -1.303 | -1.126 |
| H | 1 | -1.204 | 0.042 | 92.526 | -1.287 | -1.120 |
| C | 2 | -1.134 | 0.050 | 169.262 | -1.231 | -1.036 |
| H | 2 | -1.117 | 0.050 | 172.929 | -1.215 | -1.019 |
| C | 3 | -1.186 | 0.047 | 143.129 | -1.279 | -1.092 |
| H | 3 | -1.195 | 0.048 | 157.323 | -1.290 | -1.100 |
| Partner | Batch |  |  |  |  |  |
| C | 1 | -1.446 | 0.053 | 59.658 | -1.552 | -1.341 |
| H | 1 | -1.314 | 0.052 | 55.732 | -1.418 | -1.211 |
| C | 2 | -1.137 | 0.056 | 65.088 | -1.249 | -1.024 |
| H | 2 | -1.102 | 0.056 | 66.706 | -1.214 | -0.990 |
| Partner | Treatment |  |  |  |  |  |
| C | H | -1.241 | 0.056 | 69.082 | -1.353 | -1.129 |
| H | H | -1.105 | 0.056 | 68.812 | -1.216 | -0.994 |
| C | H+ | -1.342 | 0.053 | 57.403 | -1.448 | -1.237 |
| H | H+ | -1.311 | 0.053 | 56.159 | -1.416 | -1.206 |
| Treatment | Batch |  |  |  |  |  |
| H | 1 | -1.375 | 0.068 | 46.746 | -1.512 | -1.237 |
| H+ | 1 | -1.386 | 0.073 | 50.125 | -1.532 | -1.239 |
| H | 2 | -0.971 | 0.081 | 63.407 | -1.133 | -0.809 |
| H+ | 2 | -1.268 | 0.070 | 45.241 | -1.408 | -1.127 |
| Treatment | Phase of trial | |  |  |  |  |
| H | -1 | -1.298 | 0.068 | 147.175 | -1.433 | -1.164 |
| H+ | -1 | -1.490 | 0.062 | 108.741 | -1.613 | -1.366 |
| H | 0 | -1.311 | 0.063 | 112.239 | -1.437 | -1.186 |
| H+ | 0 | -1.348 | 0.056 | 73.494 | -1.459 | -1.237 |
| H | 1 | -1.157 | 0.057 | 73.946 | -1.269 | -1.044 |
| H+ | 1 | -1.261 | 0.055 | 65.900 | -1.370 | -1.153 |
| H | 2 | -1.021 | 0.062 | 102.837 | -1.143 | -0.899 |
| H+ | 2 | -1.230 | 0.059 | 87.948 | -1.346 | -1.113 |
| H | 3 | -1.077 | 0.060 | 95.992 | -1.196 | -0.957 |
| H+ | 3 | -1.304 | 0.057 | 77.183 | -1.417 | -1.192 |

**Table S3: Post hoc trials on models following significant interactions or single effect of one or more explanatory variable(s)**. Each model is indicated, all post hoc trials were computed using the ’lsmeans’ function with Tukey correction for multiple testing. Significant p values indicated (***: p<0.001, **: p<0.01, *: p<0.05, • :p<0.10).

| **Contrast** | **Estimate** | **SE** | **DF** | **T.ratio** | **P.value** |  | **Contrast** | **Estimate** | **SE** | **DF** | **T.ratio** | **P.value** |
| --- | --- | --- | --- | --- | --- | --- | --- | --- | --- | --- | --- | --- |
| ***model 1 : behavioural Score 1 (LD1)*** | | | | | |  | ***model 2 : behavioural Score 2 (LD2)*** | | | | | |
| Partner: Phase of trial interaction | | | | | |  | Partner: Phase of trial interaction | | | | | |
| C,-1 - H,-1 | -1.505 | 0.102 | 966.348 | -14.742 | <0.001*** |  | C,-1 - H,-1 | 0.113 | 0.102 | 967.064 | 1.105 | 0.984 |
| C,-1 - C,0 | 0.759 | 0.104 | 968.786 | 7.331 | <0.001*** |  | C,-1 - C,0 | -0.312 | 0.104 | 970.812 | -3.005 | 0.081• |
| C,-1 - H,0 | -0.889 | 0.103 | 968.908 | -8.594 | <0.001*** |  | C,-1 - H,0 | -1.055 | 0.104 | 971.017 | -10.187 | <0.001*** |
| C,-1 - C,1 | 0.367 | 0.144 | 966.275 | 2.543 | 0.247 |  | C,-1 - C,1 | -0.299 | 0.145 | 966.940 | -2.068 | 0.550 |
| C,-1 - H,1 | -1.127 | 0.144 | 967.465 | -7.809 | <0.001*** |  | C,-1 - H,1 | -0.128 | 0.145 | 969.040 | -0.887 | 0.997 |
| C,-1 - C,2 | 0.448 | 0.144 | 966.275 | 3.104 | 0.061• |  | C,-1 - C,2 | -0.389 | 0.145 | 966.940 | -2.687 | 0.180 |
| C,-1 - H,2 | -1.451 | 0.144 | 967.465 | -10.053 | <0.001*** |  | C,-1 - H,2 | -0.195 | 0.145 | 969.040 | -1.350 | 0.942 |
| C,-1 - C,3 | 0.742 | 0.144 | 966.275 | 5.140 | <0.001*** |  | C,-1 - C,3 | -0.527 | 0.145 | 966.940 | -3.645 | 0.010* |
| C,-1 - H,3 | -1.444 | 0.144 | 967.465 | -9.999 | <0.001*** |  | C,-1 - H,3 | -0.349 | 0.145 | 969.040 | -2.414 | 0.319 |
| H,-1 - C,0 | 2.265 | 0.103 | 968.923 | 21.899 | <0.001*** |  | H,-1 - C,0 | -0.425 | 0.104 | 971.041 | -4.101 | 0.002** |
| H,-1 - H,0 | 0.617 | 0.103 | 968.767 | 5.973 | <0.001*** |  | H,-1 - H,0 | -1.168 | 0.103 | 970.782 | -11.294 | <0.001*** |
| H,-1 - C,1 | 1.872 | 0.144 | 966.205 | 12.984 | <0.001*** |  | H,-1 - C,1 | -0.412 | 0.145 | 966.822 | -2.853 | 0.121 |
| H,-1 - H,1 | 0.378 | 0.144 | 967.402 | 2.621 | 0.209 |  | H,-1 - H,1 | -0.242 | 0.145 | 968.932 | -1.671 | 0.812 |
| H,-1 - C,2 | 1.953 | 0.144 | 966.205 | 13.545 | <0.001*** |  | H,-1 - C,2 | -0.502 | 0.145 | 966.822 | -3.472 | 0.019* |
| H,-1 - H,2 | 0.054 | 0.144 | 967.402 | 0.375 | 1.000 |  | H,-1 - H,2 | -0.308 | 0.145 | 968.932 | -2.134 | 0.504 |
| H,-1 - C,3 | 2.247 | 0.144 | 966.205 | 15.583 | <0.001*** |  | H,-1 - C,3 | -0.640 | 0.145 | 966.822 | -4.431 | <0.001*** |
| H,-1 - H,3 | 0.062 | 0.144 | 967.402 | 0.429 | 1.000 |  | H,-1 - H,3 | -0.462 | 0.145 | 968.932 | -3.198 | 0.046* |
| C,0 - H,0 | -1.648 | 0.105 | 966.346 | -15.765 | <0.001*** |  | C,0 - H,0 | -0.744 | 0.105 | 967.060 | -7.097 | <0.001*** |
| C,0 - C,1 | -0.392 | 0.145 | 967.590 | -2.701 | 0.175 |  | C,0 - C,1 | 0.013 | 0.146 | 968.976 | 0.086 | 1.000 |
| C,0 - H,1 | -1.886 | 0.145 | 968.907 | -12.984 | <0.001*** |  | C,0 - H,1 | 0.183 | 0.146 | 971.225 | 1.260 | 0.962 |
| C,0 - C,2 | -0.311 | 0.145 | 967.590 | -2.143 | 0.497 |  | C,0 - C,2 | -0.077 | 0.146 | 968.976 | -0.529 | 1.000 |
| C,0 - H,2 | -2.210 | 0.145 | 968.907 | -15.215 | <0.001*** |  | C,0 - H,2 | 0.116 | 0.146 | 971.225 | 0.800 | 0.999 |
| C,0 - C,3 | -0.017 | 0.145 | 967.590 | -0.120 | 1.000 |  | C,0 - C,3 | -0.216 | 0.146 | 968.976 | -1.481 | 0.900 |
| C,0 - H,3 | -2.203 | 0.145 | 968.907 | -15.161 | <0.001*** |  | C,0 - H,3 | -0.037 | 0.146 | 971.225 | -0.257 | 1.000 |
| H,0 - C,1 | 1.256 | 0.145 | 967.509 | 8.652 | <0.001*** |  | H,0 - C,1 | 0.756 | 0.145 | 968.843 | 5.199 | <0.001*** |
| H,0 - H,1 | -0.239 | 0.145 | 968.824 | -1.644 | 0.826 |  | H,0 - H,1 | 0.927 | 0.145 | 971.091 | 6.373 | <0.001*** |
| H,0 - C,2 | 1.336 | 0.145 | 967.509 | 9.210 | <0.001*** |  | H,0 - C,2 | 0.667 | 0.145 | 968.843 | 4.584 | <0.001*** |
| H,0 - H,2 | -0.563 | 0.145 | 968.824 | -3.876 | 0.004** |  | H,0 - H,2 | 0.860 | 0.145 | 971.091 | 5.913 | <0.001*** |
| H,0 - C,3 | 1.630 | 0.145 | 967.509 | 11.235 | <0.001*** |  | H,0 - C,3 | 0.528 | 0.145 | 968.843 | 3.631 | 0.011* |
| H,0 - H,3 | -0.555 | 0.145 | 968.824 | -3.823 | 0.005** |  | H,0 - H,3 | 0.706 | 0.145 | 971.091 | 4.855 | <0.001*** |
| C,1 - H,1 | -1.494 | 0.177 | 967.001 | -8.458 | <0.001*** |  | C,1 - H,1 | 0.171 | 0.177 | 968.229 | 0.965 | 0.994 |
| C,1 - C,2 | 0.081 | 0.177 | 966.205 | 0.458 | 1.000 |  | C,1 - C,2 | -0.090 | 0.177 | 966.822 | -0.506 | 1.000 |
| C,1 - H,2 | -1.818 | 0.177 | 967.001 | -10.292 | <0.001*** |  | C,1 - H,2 | 0.104 | 0.177 | 968.229 | 0.587 | 1.000 |
| C,1 - C,3 | 0.375 | 0.177 | 966.205 | 2.122 | 0.512 |  | C,1 - C,3 | -0.228 | 0.177 | 966.822 | -1.288 | 0.956 |
| C,1 - H,3 | -1.810 | 0.177 | 967.001 | -10.248 | <0.001*** |  | C,1 - H,3 | -0.050 | 0.177 | 968.229 | -0.282 | 1.000 |
| H,1 - C,2 | 1.575 | 0.177 | 967.001 | 8.916 | <0.001*** |  | H,1 - C,2 | -0.260 | 0.177 | 968.229 | -1.470 | 0.904 |
| H,1 - H,2 | -0.324 | 0.177 | 966.205 | -1.835 | 0.713 |  | H,1 - H,2 | -0.067 | 0.177 | 966.822 | -0.378 | 1.000 |
| H,1 - C,3 | 1.869 | 0.177 | 967.001 | 10.580 | <0.001*** |  | H,1 - C,3 | -0.399 | 0.177 | 968.229 | -2.253 | 0.421 |
| H,1 - H,3 | -0.316 | 0.177 | 966.205 | -1.791 | 0.741 |  | H,1 - H,3 | -0.221 | 0.177 | 966.822 | -1.247 | 0.964 |
| C,2 - H,2 | -1.899 | 0.177 | 967.001 | -10.751 | <0.001*** |  | C,2 - H,2 | 0.193 | 0.177 | 968.229 | 1.092 | 0.985 |
| C,2 - C,3 | 0.294 | 0.177 | 966.205 | 1.664 | 0.816 |  | C,2 - C,3 | -0.139 | 0.177 | 966.822 | -0.783 | 0.999 |
| C,2 - H,3 | -1.891 | 0.177 | 967.001 | -10.707 | <0.001*** |  | C,2 - H,3 | 0.040 | 0.177 | 968.229 | 0.223 | 1.000 |
| H,2 - C,3 | 2.193 | 0.177 | 967.001 | 12.414 | <0.001*** |  | H,2 - C,3 | -0.332 | 0.177 | 968.229 | -1.875 | 0.686 |
| H,2 - H,3 | 0.008 | 0.177 | 966.205 | 0.044 | 1.000 |  | H,2 - H,3 | -0.154 | 0.177 | 966.822 | -0.869 | 0.997 |
| C,3 - H,3 | -2.185 | 0.177 | 967.001 | -12.370 | <0.001*** |  | C,3 - H,3 | 0.178 | 0.177 | 968.229 | 1.006 | 0.992 |
| ***model 3 : log( Total time spent near upcoming partner's door +1)*** | | | | | |  | ***model 3 : log( Total time spent near upcoming partner's door +1)*** | | | | | |
| Partner: Trial (3 levels of factor) | | | | | |  | Treatment: Batch interaction | | | | | |
| beginning,C - end,C | -0.570 | 0.125 | 189.673 | -4.546 | <0.001*** |  | H,1 - H+,1 | -0.014 | 0.113 | 134.234 | -0.128 | 0.999 |
| beginning,C - middle,C | 0.008 | 0.140 | 527.352 | 0.057 | 1.000 |  | H,1 - H,2 | -0.107 | 0.106 | 108.450 | -1.014 | 0.742 |
| beginning,C - beginning,H | 0.913 | 0.130 | 639.411 | 7.021 | <0.001*** |  | H,1 - H+,2 | 0.181 | 0.108 | 133.567 | 1.677 | 0.340 |
| beginning,C - end,H | 0.814 | 0.125 | 189.230 | 6.498 | <0.001*** |  | H+,1 - H,2 | -0.093 | 0.104 | 113.122 | -0.893 | 0.808 |
| beginning,C - middle,H | 0.880 | 0.141 | 528.253 | 6.250 | <0.001*** |  | H+,1 - H+,2 | 0.195 | 0.105 | 91.903 | 1.866 | 0.250 |
| end,C - middle,C | 0.578 | 0.138 | 197.543 | 4.187 | 0.001** |  | H,2 - H+,2 | 0.288 | 0.098 | 108.150 | 2.932 | 0.021* |
| end,C - beginning,H | 1.483 | 0.124 | 182.762 | 11.980 | <0.001*** |  |  |  |  |  |  |  |
| end,C - end,H | 1.384 | 0.103 | 615.691 | 13.438 | <0.001*** |  |  |  |  |  |  |  |
| end,C - middle,H | 1.450 | 0.138 | 197.204 | 10.512 | <0.001*** |  |  |  |  |  |  |  |
| middle,C - beginning,H | 0.905 | 0.139 | 524.240 | 6.506 | <0.001*** |  |  |  |  |  |  |  |
| middle,C - end,H | 0.806 | 0.137 | 195.104 | 5.862 | <0.001*** |  |  |  |  |  |  |  |
| middle,C - middle,H | 0.872 | 0.148 | 616.184 | 5.901 | <0.001*** |  |  |  |  |  |  |  |
| beginning,H - end,H | -0.099 | 0.124 | 181.829 | -0.802 | 0.967 |  |  |  |  |  |  |  |
| beginning,H - middle,H | -0.033 | 0.140 | 527.302 | -0.236 | 1.000 |  |  |  |  |  |  |  |
| end,H - middle,H | 0.066 | 0.138 | 199.068 | 0.478 | 0.997 |  |  |  |  |  |  |  |
| ***model 5 : Spectral acoustic Score (LD1)*** | | | | | |  | ***model 5 : Spectral acoustic Score (LD1)*** | | | | | |
| Partner: Phase of trial interaction | | | | | |  | Treatment: Phase of trial interaction | | | | | |
| C,-1 - H,-1 | 0.011 | 0.115 | 2217.203 | 0.098 | 1.000 |  | H,-1 - H+,-1 | -0.525 | 0.172 | 142.068 | -3.055 | 0.078• |
| C,-1 - C,0 | -0.408 | 0.103 | 2209.822 | -3.965 | 0.003** |  | H,-1 - H,0 | -0.203 | 0.112 | 2215.443 | -1.804 | 0.733 |
| C,-1 - H,0 | 0.424 | 0.105 | 2216.153 | 4.043 | 0.002** |  | H,-1 - H+,0 | -0.317 | 0.164 | 118.129 | -1.935 | 0.645 |
| C,-1 - C,1 | 0.064 | 0.097 | 2208.760 | 0.664 | 1.000 |  | H,-1 - H,1 | -0.121 | 0.101 | 2220.257 | -1.200 | 0.973 |
| C,-1 - H,1 | 0.120 | 0.095 | 2229.751 | 1.257 | 0.963 |  | H,-1 - H+,1 | -0.232 | 0.162 | 112.282 | -1.434 | 0.914 |
| C,-1 - C,2 | 0.057 | 0.106 | 2213.284 | 0.537 | 1.000 |  | H,-1 - H,2 | -0.048 | 0.114 | 2232.977 | -0.418 | 1.000 |
| C,-1 - H,2 | 0.150 | 0.109 | 2236.493 | 1.375 | 0.935 |  | H,-1 - H+,2 | -0.282 | 0.167 | 128.115 | -1.687 | 0.800 |
| C,-1 - C,3 | 0.060 | 0.104 | 2222.636 | 0.576 | 1.000 |  | H,-1 - H,3 | -0.045 | 0.113 | 2237.855 | -0.401 | 1.000 |
| C,-1 - H,3 | -0.024 | 0.107 | 2241.714 | -0.227 | 1.000 |  | H,-1 - H+,3 | -0.456 | 0.164 | 120.742 | -2.772 | 0.158 |
| H,-1 - C,0 | -0.419 | 0.104 | 2217.079 | -4.023 | 0.002** |  | H+,-1 - H,0 | 0.322 | 0.165 | 121.336 | 1.952 | 0.634 |
| H,-1 - H,0 | 0.413 | 0.105 | 2218.536 | 3.951 | 0.003** |  | H+,-1 - H+,0 | 0.208 | 0.094 | 2214.486 | 2.207 | 0.452 |
| H,-1 - C,1 | 0.053 | 0.098 | 2219.878 | 0.540 | 1.000 |  | H+,-1 - H,1 | 0.404 | 0.156 | 96.944 | 2.597 | 0.233 |
| H,-1 - H,1 | 0.108 | 0.094 | 2224.328 | 1.153 | 0.979 |  | H+,-1 - H+,1 | 0.293 | 0.090 | 2210.346 | 3.255 | 0.038* |
| H,-1 - C,2 | 0.046 | 0.108 | 2227.904 | 0.425 | 1.000 |  | H+,-1 - H,2 | 0.478 | 0.163 | 115.836 | 2.934 | 0.108 |
| H,-1 - H,2 | 0.139 | 0.108 | 2230.613 | 1.287 | 0.957 |  | H+,-1 - H+,2 | 0.243 | 0.100 | 2212.764 | 2.423 | 0.313 |
| H,-1 - C,3 | 0.048 | 0.104 | 2230.920 | 0.463 | 1.000 |  | H+,-1 - H,3 | 0.480 | 0.161 | 111.828 | 2.987 | 0.095• |
| H,-1 - H,3 | -0.036 | 0.106 | 2239.583 | -0.335 | 1.000 |  | H+,-1 - H+,3 | 0.069 | 0.097 | 2216.499 | 0.714 | 0.999 |
| C,0 - H,0 | 0.832 | 0.092 | 2213.600 | 9.005 | <0.001*** |  | H,0 - H+,0 | -0.114 | 0.157 | 99.103 | -0.729 | 0.999 |
| C,0 - C,1 | 0.472 | 0.083 | 2209.145 | 5.675 | <0.001*** |  | H,0 - H,1 | 0.082 | 0.090 | 2226.312 | 0.917 | 0.996 |
| C,0 - H,1 | 0.527 | 0.081 | 2234.726 | 6.502 | <0.001*** |  | H,0 - H+,1 | -0.029 | 0.155 | 93.729 | -0.189 | 1.000 |
| C,0 - C,2 | 0.465 | 0.095 | 2222.188 | 4.903 | <0.001*** |  | H,0 - H,2 | 0.155 | 0.103 | 2231.712 | 1.503 | 0.891 |
| C,0 - H,2 | 0.558 | 0.096 | 2236.855 | 5.782 | <0.001*** |  | H,0 - H+,2 | -0.079 | 0.160 | 108.272 | -0.494 | 1.000 |
| C,0 - C,3 | 0.467 | 0.091 | 2223.392 | 5.147 | <0.001*** |  | H,0 - H,3 | 0.158 | 0.102 | 2238.722 | 1.540 | 0.876 |
| C,0 - H,3 | 0.383 | 0.094 | 2244.946 | 4.064 | 0.002** |  | H,0 - H+,3 | -0.253 | 0.157 | 101.416 | -1.608 | 0.841 |
| H,0 - C,1 | -0.360 | 0.086 | 2224.225 | -4.200 | 0.001** |  | H+,0 - H,1 | 0.196 | 0.147 | 76.927 | 1.338 | 0.941 |
| H,0 - H,1 | -0.305 | 0.081 | 2231.749 | -3.744 | 0.007** |  | H+,0 - H+,1 | 0.085 | 0.075 | 2223.071 | 1.134 | 0.981 |
| H,0 - C,2 | -0.367 | 0.096 | 2225.966 | -3.842 | 0.005** |  | H+,0 - H,2 | 0.269 | 0.154 | 94.007 | 1.747 | 0.765 |
| H,0 - H,2 | -0.274 | 0.096 | 2231.261 | -2.850 | 0.121 |  | H+,0 - H+,2 | 0.035 | 0.086 | 2217.711 | 0.405 | 1.000 |
| H,0 - C,3 | -0.365 | 0.092 | 2228.687 | -3.967 | 0.003** |  | H+,0 - H,3 | 0.272 | 0.152 | 90.068 | 1.788 | 0.741 |
| H,0 - H,3 | -0.449 | 0.095 | 2242.615 | -4.723 | <0.001*** |  | H+,0 - H+,3 | -0.139 | 0.082 | 2213.225 | -1.695 | 0.799 |
| C,1 - H,1 | 0.055 | 0.073 | 2240.836 | 0.761 | 0.999 |  | H,1 - H+,1 | -0.111 | 0.145 | 72.230 | -0.770 | 0.999 |
| C,1 - C,2 | -0.007 | 0.087 | 2214.775 | -0.083 | 1.000 |  | H,1 - H,2 | 0.073 | 0.088 | 2224.569 | 0.835 | 0.998 |
| C,1 - H,2 | 0.086 | 0.090 | 2242.541 | 0.953 | 0.995 |  | H,1 - H+,2 | -0.161 | 0.150 | 85.153 | -1.072 | 0.986 |
| C,1 - C,3 | -0.005 | 0.083 | 2224.133 | -0.055 | 1.000 |  | H,1 - H,3 | 0.075 | 0.086 | 2233.648 | 0.876 | 0.997 |
| C,1 - H,3 | -0.089 | 0.087 | 2246.887 | -1.016 | 0.991 |  | H,1 - H+,3 | -0.335 | 0.148 | 78.997 | -2.272 | 0.419 |
| H,1 - C,2 | -0.063 | 0.085 | 2243.554 | -0.735 | 0.999 |  | H+,1 - H,2 | 0.185 | 0.152 | 88.741 | 1.213 | 0.968 |
| H,1 - H,2 | 0.030 | 0.082 | 2218.897 | 0.369 | 1.000 |  | H+,1 - H+,2 | -0.050 | 0.082 | 2211.875 | -0.613 | 1.000 |
| H,1 - C,3 | -0.060 | 0.081 | 2246.462 | -0.737 | 0.999 |  | H+,1 - H,3 | 0.187 | 0.150 | 85.030 | 1.246 | 0.962 |
| H,1 - H,3 | -0.144 | 0.080 | 2235.714 | -1.789 | 0.742 |  | H+,1 - H+,3 | -0.224 | 0.077 | 2216.851 | -2.897 | 0.107 |
| C,2 - H,2 | 0.093 | 0.100 | 2243.084 | 0.928 | 0.996 |  | H,2 - H+,2 | -0.234 | 0.158 | 102.963 | -1.487 | 0.894 |
| C,2 - C,3 | 0.003 | 0.092 | 2210.153 | 0.029 | 1.000 |  | H,2 - H,3 | 0.002 | 0.098 | 2216.547 | 0.023 | 1.000 |
| C,2 - H,3 | -0.081 | 0.098 | 2246.993 | -0.829 | 0.998 |  | H,2 - H+,3 | -0.408 | 0.155 | 96.229 | -2.636 | 0.216 |
| H,2 - C,3 | -0.090 | 0.097 | 2245.410 | -0.933 | 0.995 |  | H+,2 - H,3 | 0.237 | 0.156 | 99.241 | 1.521 | 0.880 |
| H,2 - H,3 | -0.174 | 0.094 | 2220.675 | -1.853 | 0.701 |  | H+,2 - H+,3 | -0.174 | 0.088 | 2211.712 | -1.974 | 0.617 |
| C,3 - H,3 | -0.084 | 0.095 | 2245.497 | -0.888 | 0.997 |  | H,3 - H+,3 | -0.411 | 0.153 | 92.588 | -2.688 | 0.194 |
| Partner: Treatment interaction | | | | | |  | Partner: Batch interaction | | | | | |
| C,H - H,H | 0.281 | 0.070 | 2227.517 | 4.018 | <0.001*** |  | C,1 - H,1 | 0.341 | 0.060 | 2229.718 | 5.706 | <0.001*** |
| C,H - C,H+ | -0.179 | 0.141 | 64.437 | -1.273 | 0.583 |  | C,1 - C,2 | 0.202 | 0.140 | 62.744 | 1.448 | 0.475 |
| C,H - H,H+ | -0.098 | 0.140 | 63.322 | -0.696 | 0.898 |  | C,1 - H,2 | 0.224 | 0.140 | 64.074 | 1.604 | 0.384 |
| H,H - C,H+ | -0.461 | 0.139 | 63.808 | -3.303 | 0.008** |  | H,1 - C,2 | -0.139 | 0.139 | 61.102 | -1.001 | 0.750 |
| H,H - H,H+ | -0.379 | 0.140 | 63.571 | -2.717 | 0.04* |  | H,1 - H,2 | -0.117 | 0.138 | 61.049 | -0.846 | 0.832 |
| C,H+ - H,H+ | 0.082 | 0.060 | 2245.888 | 1.369 | 0.519 |  | C,2 - H,2 | 0.022 | 0.067 | 2244.870 | 0.327 | 0.988 |
| ***model 6 : log( Vocalisation duration)*** | | | | | |  | ***model 6 : log( Vocalisation duration)*** | | | | | |
| Partner: Phase of trial interaction | | | | | |  | Treatment: Phase of trial interaction | | | | | |
| C,-1 - H,-1 | -0.008 | 0.059 | 2214.509 | -0.141 | 1.000 |  | H,-1 - H+,-1 | 0.191 | 0.092 | 127.492 | 2.074 | 0.549 |
| C,-1 - C,0 | 0.127 | 0.052 | 2207.760 | 2.418 | 0.315 |  | H,-1 - H,0 | 0.013 | 0.057 | 2213.434 | 0.227 | 1.000 |
| C,-1 - H,0 | -0.263 | 0.053 | 2213.425 | -4.928 | <0.001*** |  | H,-1 - H+,0 | 0.050 | 0.088 | 107.926 | 0.563 | 1.000 |
| C,-1 - C,1 | -0.183 | 0.049 | 2206.907 | -3.723 | 0.008** |  | H,-1 - H,1 | -0.142 | 0.051 | 2217.319 | -2.765 | 0.149 |
| C,-1 - H,1 | -0.195 | 0.048 | 2225.623 | -4.017 | 0.002 |  | H,-1 - H+,1 | -0.037 | 0.087 | 103.187 | -0.423 | 1.000 |
| C,-1 - C,2 | -0.264 | 0.054 | 2211.248 | -4.884 | <0.001*** |  | H,-1 - H,2 | -0.277 | 0.058 | 2229.053 | -4.791 | <0.001*** |
| C,-1 - H,2 | -0.281 | 0.056 | 2232.014 | -5.069 | <0.001*** |  | H,-1 - H+,2 | -0.069 | 0.090 | 116.076 | -0.765 | 0.999 |
| C,-1 - C,3 | -0.212 | 0.053 | 2219.543 | -4.022 | 0.002** |  | H,-1 - H,3 | -0.222 | 0.058 | 2234.701 | -3.852 | 0.005** |
| C,-1 - H,3 | -0.203 | 0.054 | 2238.763 | -3.728 | 0.008** |  | H,-1 - H+,3 | 0.006 | 0.089 | 109.947 | 0.066 | 1.000 |
| H,-1 - C,0 | 0.135 | 0.053 | 2214.689 | 2.543 | 0.246 |  | H+,-1 - H,0 | -0.178 | 0.089 | 110.503 | -2.005 | 0.597 |
| H,-1 - H,0 | -0.255 | 0.053 | 2215.989 | -4.793 | <0.001*** |  | H+,-1 - H+,0 | -0.141 | 0.048 | 2211.834 | -2.947 | 0.094 |
| H,-1 - C,1 | -0.175 | 0.050 | 2217.356 | -3.512 | 0.016* |  | H+,-1 - H,1 | -0.333 | 0.084 | 90.422 | -3.942 | 0.006** |
| H,-1 - H,1 | -0.186 | 0.048 | 2220.846 | -3.897 | 0.004** |  | H+,-1 - H+,1 | -0.228 | 0.046 | 2208.007 | -4.976 | <0.001*** |
| H,-1 - C,2 | -0.256 | 0.055 | 2224.663 | -4.675 | <0.001*** |  | H+,-1 - H,2 | -0.469 | 0.088 | 105.757 | -5.342 | <0.001*** |
| H,-1 - H,2 | -0.273 | 0.055 | 2226.726 | -4.983 | <0.001*** |  | H+,-1 - H+,2 | -0.260 | 0.051 | 2210.669 | -5.087 | <0.001*** |
| H,-1 - C,3 | -0.204 | 0.053 | 2227.487 | -3.835 | 0.005** |  | H+,-1 - H,3 | -0.413 | 0.087 | 102.286 | -4.763 | <0.001*** |
| H,-1 - H,3 | -0.195 | 0.054 | 2236.850 | -3.592 | 0.012* |  | H+,-1 - H+,3 | -0.185 | 0.049 | 2213.912 | -3.751 | 0.007** |
| C,0 - H,0 | -0.390 | 0.047 | 2211.157 | -8.290 | <0.001*** |  | H,0 - H+,0 | 0.037 | 0.085 | 92.223 | 0.433 | 1.000 |
| C,0 - C,1 | -0.310 | 0.042 | 2207.577 | -7.321 | <0.001*** |  | H,0 - H,1 | -0.155 | 0.046 | 2223.429 | -3.383 | 0.025* |
| C,0 - H,1 | -0.321 | 0.041 | 2230.690 | -7.774 | <0.001*** |  | H,0 - H+,1 | -0.050 | 0.084 | 87.833 | -0.595 | 1.000 |
| C,0 - C,2 | -0.391 | 0.048 | 2219.303 | -8.097 | <0.001*** |  | H,0 - H,2 | -0.290 | 0.053 | 2227.936 | -5.520 | <0.001*** |
| C,0 - H,2 | -0.408 | 0.049 | 2232.506 | -8.304 | <0.001*** |  | H,0 - H+,2 | -0.082 | 0.086 | 99.763 | -0.945 | 0.994 |
| C,0 - C,3 | -0.339 | 0.046 | 2220.125 | -7.325 | <0.001*** |  | H,0 - H,3 | -0.235 | 0.052 | 2235.931 | -4.501 | <0.001*** |
| C,0 - H,3 | -0.330 | 0.048 | 2242.830 | -6.858 | <0.001*** |  | H,0 - H+,3 | -0.007 | 0.085 | 94.035 | -0.084 | 1.000 |
| H,0 - C,1 | 0.080 | 0.044 | 2221.179 | 1.833 | 0.714 |  | H+,0 - H,1 | -0.191 | 0.080 | 73.783 | -2.387 | 0.349 |
| H,0 - H,1 | 0.069 | 0.041 | 2227.913 | 1.660 | 0.818 |  | H+,0 - H+,1 | -0.087 | 0.038 | 2219.435 | -2.270 | 0.409 |
| H,0 - C,2 | -0.001 | 0.049 | 2223.051 | -0.019 | 1.000 |  | H+,0 - H,2 | -0.327 | 0.084 | 87.806 | -3.912 | 0.007** |
| H,0 - H,2 | -0.018 | 0.049 | 2227.028 | -0.368 | 1.000 |  | H+,0 - H+,2 | -0.118 | 0.044 | 2215.176 | -2.689 | 0.179 |
| H,0 - C,3 | 0.051 | 0.047 | 2225.491 | 1.093 | 0.985 |  | H+,0 - H,3 | -0.271 | 0.083 | 84.400 | -3.289 | 0.045* |
| H,0 - H,3 | 0.060 | 0.048 | 2240.114 | 1.243 | 0.965 |  | H+,0 - H+,3 | -0.044 | 0.042 | 2210.750 | -1.052 | 0.989 |
| C,1 - H,1 | -0.011 | 0.037 | 2237.103 | -0.302 | 1.000 |  | H,1 - H+,1 | 0.105 | 0.079 | 69.890 | 1.323 | 0.945 |
| C,1 - C,2 | -0.081 | 0.044 | 2212.857 | -1.831 | 0.716 |  | H,1 - H,2 | -0.136 | 0.045 | 2221.435 | -3.051 | 0.070 |
| C,1 - H,2 | -0.098 | 0.046 | 2238.764 | -2.141 | 0.498 |  | H,1 - H+,2 | 0.073 | 0.082 | 80.634 | 0.890 | 0.996 |
| C,1 - C,3 | -0.029 | 0.042 | 2221.238 | -0.681 | 1.000 |  | H,1 - H,3 | -0.080 | 0.044 | 2231.189 | -1.830 | 0.716 |
| C,1 - H,3 | -0.020 | 0.044 | 2245.906 | -0.446 | 1.000 |  | H,1 - H+,3 | 0.147 | 0.080 | 75.439 | 1.832 | 0.713 |
| H,1 - C,2 | -0.070 | 0.043 | 2240.324 | -1.608 | 0.844 |  | H+,1 - H,2 | -0.241 | 0.083 | 83.508 | -2.911 | 0.119 |
| H,1 - H,2 | -0.087 | 0.042 | 2215.873 | -2.076 | 0.545 |  | H+,1 - H+,2 | -0.032 | 0.042 | 2210.220 | -0.766 | 0.999 |
| H,1 - C,3 | -0.018 | 0.041 | 2244.356 | -0.426 | 1.000 |  | H+,1 - H,3 | -0.185 | 0.082 | 80.281 | -2.266 | 0.422 |
| H,1 - H,3 | -0.009 | 0.041 | 2233.590 | -0.211 | 1.000 |  | H+,1 - H+,3 | 0.043 | 0.039 | 2214.573 | 1.087 | 0.986 |
| C,2 - H,2 | -0.017 | 0.051 | 2239.442 | -0.336 | 1.000 |  | H,2 - H+,2 | 0.209 | 0.085 | 95.185 | 2.448 | 0.310 |
| C,2 - C,3 | 0.052 | 0.047 | 2209.017 | 1.110 | 0.984 |  | H,2 - H,3 | 0.056 | 0.050 | 2215.502 | 1.118 | 0.983 |
| C,2 - H,3 | 0.061 | 0.050 | 2246.484 | 1.221 | 0.969 |  | H,2 - H+,3 | 0.283 | 0.084 | 89.562 | 3.375 | 0.035* |
| H,2 - C,3 | 0.069 | 0.049 | 2242.411 | 1.405 | 0.926 |  | H+,2 - H,3 | -0.153 | 0.084 | 91.950 | -1.816 | 0.723 |
| H,2 - H,3 | 0.078 | 0.048 | 2219.511 | 1.634 | 0.831 |  | H+,2 - H+,3 | 0.075 | 0.045 | 2210.373 | 1.663 | 0.816 |
| C,3 - H,3 | 0.009 | 0.048 | 2246.911 | 0.187 | 1.000 |  | H,3 - H+,3 | 0.228 | 0.083 | 86.395 | 2.745 | 0.173 |
| Partner:Treatment interaction | |  |  |  |  |  | Partner:Batch interaction | | | | | |
| C,H - H,H | -0.136 | 0.036 | 2238.191 | -3.803 | 0.001** |  | C,1 - H,1 | -0.132 | 0.031 | 2241.341 | -4.329 | <0.001*** |
| C,H - C,H+ | 0.101 | 0.077 | 63.411 | 1.311 | 0.559 |  | C,1 - C,2 | -0.310 | 0.077 | 61.934 | -4.033 | 0.001** |
| C,H - H,H+ | 0.070 | 0.077 | 62.477 | 0.912 | 0.798 |  | C,1 - H,2 | -0.344 | 0.077 | 62.924 | -4.488 | <0.001*** |
| H,H - C,H+ | 0.237 | 0.077 | 62.634 | 3.097 | 0.015* |  | H,1 - C,2 | -0.177 | 0.076 | 60.532 | -2.324 | 0.104 |
| H,H - H,H+ | 0.206 | 0.077 | 62.478 | 2.691 | 0.044* |  | H,1 - H,2 | -0.212 | 0.076 | 60.360 | -2.795 | 0.034* |
| C,H+ - H,H+ | -0.031 | 0.030 | 2246.941 | -1.021 | 0.737 |  | C,2 - H,2 | -0.035 | 0.034 | 2246.804 | -1.020 | 0.738 |
| Treatment:Batch interaction |  |  |  |  |  |  |  |  |  |  |  |  |
| H,1 - H+,1 | 0.011 | 0.100 | 48.467 | 0.108 | 1.000 |  |  |  |  |  |  |  |
| H,1 - H,2 | -0.404 | 0.105 | 54.967 | -3.831 | 0.002** |  |  |  |  |  |  |  |
| H,1 - H+,2 | -0.107 | 0.098 | 45.975 | -1.095 | 0.694 |  |  |  |  |  |  |  |
| H+,1 - H,2 | -0.415 | 0.109 | 56.833 | -3.822 | 0.002** |  |  |  |  |  |  |  |
| H+,1 - H+,2 | -0.118 | 0.101 | 47.419 | -1.171 | 0.648 |  |  |  |  |  |  |  |
| H,2 - H+,2 | 0.297 | 0.107 | 54.466 | 2.780 | 0.036* |  |  |  |  |  |  |  |

## Data composition tables

**Table S4: Acoustic values for acoustic proxies per group (Treatment: Partner: Phase of trial)**. The number of vocalisations per group is indicated and mean, standard deviation (sd), standard error (se) and 95% confidence interval (ci) are indicated for the vocalisation duration and all the spectral parameters used to build the spectral acoustic score (LD1)

|  |  |  |  | **Grunt duration (s)** | | | | **Mean (Hz)** | | | | **Median (Hz)** | | | | **Mode (Hz)** | | | |
| --- | --- | --- | --- | --- | --- | --- | --- | --- | --- | --- | --- | --- | --- | --- | --- | --- | --- | --- | --- |
| **Treatment** | **Partner** | **Phase of trial** | **N** | **mean** | **sd** | **se** | **ci** | **mean** | **sd** | **se** | **ci** | **mean** | **sd** | **se** | **ci** | **mean** | **sd** | **se** | **ci** |
| H | C | -1 | 56 | 0.270 | 0.150 | 0.020 | 0.040 | 1684.124 | 327.780 | 43.801 | 87.780 | 620.722 | 348.976 | 46.634 | 93.456 | 311.146 | 43.253 | 5.780 | 11.583 |
| H | C | 0 | 86 | 0.230 | 0.166 | 0.018 | 0.036 | 1567.744 | 269.436 | 29.054 | 57.767 | 537.490 | 194.757 | 21.001 | 41.756 | 321.186 | 43.040 | 4.641 | 9.228 |
| H | C | 1 | 158 | 0.378 | 0.238 | 0.019 | 0.037 | 1603.305 | 292.058 | 23.235 | 45.893 | 515.844 | 182.099 | 14.487 | 28.615 | 301.127 | 42.061 | 3.346 | 6.609 |
| H | C | 2 | 94 | 0.437 | 0.242 | 0.025 | 0.050 | 1507.209 | 245.191 | 25.289 | 50.220 | 462.287 | 134.290 | 13.851 | 27.505 | 293.664 | 42.098 | 4.342 | 8.623 |
| H | C | 3 | 107 | 0.427 | 0.250 | 0.024 | 0.048 | 1525.283 | 294.430 | 28.464 | 56.432 | 517.038 | 231.680 | 22.397 | 44.405 | 298.408 | 41.811 | 4.042 | 8.014 |
| H | H | -1 | 63 | 0.306 | 0.185 | 0.023 | 0.047 | 1739.732 | 311.840 | 39.288 | 78.536 | 590.546 | 241.963 | 30.485 | 60.938 | 328.205 | 41.924 | 5.282 | 10.558 |
| H | H | 0 | 78 | 0.369 | 0.193 | 0.022 | 0.044 | 1653.975 | 305.643 | 34.607 | 68.912 | 554.748 | 249.115 | 28.207 | 56.167 | 300.592 | 50.770 | 5.749 | 11.447 |
| H | H | 1 | 183 | 0.389 | 0.195 | 0.014 | 0.028 | 1602.807 | 286.368 | 21.169 | 41.768 | 534.871 | 227.058 | 16.785 | 33.117 | 295.346 | 42.871 | 3.169 | 6.253 |
| H | H | 2 | 81 | 0.404 | 0.184 | 0.020 | 0.041 | 1618.200 | 292.162 | 32.462 | 64.602 | 563.397 | 240.758 | 26.751 | 53.236 | 297.924 | 43.314 | 4.813 | 9.578 |
| H | H | 3 | 83 | 0.408 | 0.181 | 0.020 | 0.039 | 1519.422 | 270.347 | 29.674 | 59.032 | 503.429 | 182.851 | 20.071 | 39.927 | 294.347 | 42.547 | 4.670 | 9.291 |
| H+ | C | -1 | 82 | 0.259 | 0.120 | 0.013 | 0.026 | 1502.188 | 264.115 | 29.167 | 58.032 | 472.045 | 129.253 | 14.274 | 28.400 | 310.480 | 49.324 | 5.447 | 10.838 |
| H+ | C | 0 | 139 | 0.246 | 0.116 | 0.010 | 0.019 | 1555.290 | 231.077 | 19.600 | 38.755 | 496.166 | 146.289 | 12.408 | 24.534 | 315.408 | 43.770 | 3.713 | 7.341 |
| H+ | C | 1 | 146 | 0.324 | 0.142 | 0.012 | 0.023 | 1587.505 | 284.500 | 23.545 | 46.537 | 503.989 | 174.373 | 14.431 | 28.523 | 302.514 | 45.484 | 3.764 | 7.440 |
| H+ | C | 2 | 93 | 0.345 | 0.174 | 0.018 | 0.036 | 1497.941 | 241.367 | 25.029 | 49.709 | 479.074 | 163.642 | 16.969 | 33.702 | 296.065 | 43.556 | 4.517 | 8.970 |
| H+ | C | 3 | 113 | 0.298 | 0.151 | 0.014 | 0.028 | 1507.152 | 292.735 | 27.538 | 54.563 | 501.177 | 191.904 | 18.053 | 35.769 | 304.580 | 44.377 | 4.175 | 8.271 |
| H+ | H | -1 | 70 | 0.243 | 0.112 | 0.013 | 0.027 | 1575.042 | 244.688 | 29.246 | 58.344 | 498.713 | 203.349 | 24.305 | 48.487 | 303.925 | 45.780 | 5.472 | 10.916 |
| H+ | H | 0 | 142 | 0.327 | 0.140 | 0.012 | 0.023 | 1555.213 | 265.097 | 22.246 | 43.980 | 496.022 | 168.004 | 14.099 | 27.872 | 299.396 | 48.115 | 4.038 | 7.982 |
| H+ | H | 1 | 247 | 0.334 | 0.157 | 0.010 | 0.020 | 1596.026 | 282.566 | 17.979 | 35.413 | 506.687 | 175.430 | 11.162 | 21.986 | 303.742 | 47.220 | 3.005 | 5.918 |
| H+ | H | 2 | 113 | 0.357 | 0.183 | 0.017 | 0.034 | 1594.982 | 307.845 | 28.960 | 57.380 | 555.247 | 235.247 | 22.130 | 43.848 | 293.222 | 47.574 | 4.475 | 8.867 |
| H+ | H | 3 | 136 | 0.336 | 0.182 | 0.016 | 0.031 | 1581.890 | 276.006 | 23.667 | 46.807 | 514.092 | 190.153 | 16.305 | 32.247 | 301.681 | 43.880 | 3.763 | 7.442 |
|  |  |  |  |  |  |  |  |  |  |  |  |  |  |  |  |  |  |  |  |
|  |  |  |  | **First Quartile Q25 (Hz)** | | | | **Third Quartile Q75 (Hz)** | | | | **Centroid (Hz)** | | | |  |  |  |  |
| **Treatment** | **Partner** | **Phase of test** | **N** | **mean** | **sd** | **se** | **ci** | **mean** | **sd** | **se** | **ci** | **mean** | **sd** | **se** | **ci** |  |  |  |  |
| H | C | -1 | 56 | 302.041 | 54.385 | 7.267 | 14.564 | 2420.879 | 819.325 | 109.487 | 219.417 | 1684.124 | 327.780 | 43.801 | 87.780 |  |  |  |  |
| H | C | 0 | 86 | 306.670 | 45.937 | 4.954 | 9.849 | 2027.396 | 699.634 | 75.443 | 150.002 | 1567.744 | 269.436 | 29.054 | 57.767 |  |  |  |  |
| H | C | 1 | 158 | 286.549 | 40.281 | 3.205 | 6.330 | 2231.075 | 753.146 | 59.917 | 118.348 | 1603.305 | 292.058 | 23.235 | 45.893 |  |  |  |  |
| H | C | 2 | 94 | 274.024 | 30.444 | 3.140 | 6.235 | 1965.396 | 663.033 | 68.387 | 135.802 | 1507.209 | 245.191 | 25.289 | 50.220 |  |  |  |  |
| H | C | 3 | 107 | 282.058 | 37.747 | 3.649 | 7.235 | 1975.064 | 778.304 | 75.241 | 149.174 | 1525.283 | 294.430 | 28.464 | 56.432 |  |  |  |  |
| H | H | -1 | 63 | 310.344 | 47.490 | 5.983 | 11.960 | 2565.358 | 763.230 | 96.158 | 192.217 | 1739.732 | 311.840 | 39.288 | 78.536 |  |  |  |  |
| H | H | 0 | 78 | 284.813 | 38.991 | 4.415 | 8.791 | 2302.733 | 750.096 | 84.932 | 169.121 | 1653.975 | 305.643 | 34.607 | 68.912 |  |  |  |  |
| H | H | 1 | 183 | 287.708 | 41.677 | 3.081 | 6.079 | 2197.610 | 731.375 | 54.065 | 106.674 | 1602.807 | 286.368 | 21.169 | 41.768 |  |  |  |  |
| H | H | 2 | 81 | 287.179 | 40.046 | 4.450 | 8.855 | 2219.726 | 695.209 | 77.245 | 153.723 | 1618.200 | 292.162 | 32.462 | 64.602 |  |  |  |  |
| H | H | 3 | 83 | 280.154 | 33.959 | 3.727 | 7.415 | 1974.527 | 661.681 | 72.629 | 144.482 | 1519.422 | 270.347 | 29.674 | 59.032 |  |  |  |  |
| H+ | C | -1 | 82 | 284.428 | 37.511 | 4.142 | 8.242 | 1986.813 | 765.804 | 84.569 | 168.266 | 1502.188 | 264.115 | 29.167 | 58.032 |  |  |  |  |
| H+ | C | 0 | 139 | 288.908 | 42.600 | 3.613 | 7.145 | 2114.444 | 645.644 | 54.763 | 108.283 | 1555.290 | 231.077 | 19.600 | 38.755 |  |  |  |  |
| H+ | C | 1 | 146 | 286.076 | 40.886 | 3.384 | 6.688 | 2213.157 | 736.747 | 60.974 | 120.512 | 1587.505 | 284.500 | 23.545 | 46.537 |  |  |  |  |
| H+ | C | 2 | 93 | 272.717 | 30.536 | 3.166 | 6.289 | 1991.540 | 649.380 | 67.338 | 133.738 | 1497.941 | 241.367 | 25.029 | 49.709 |  |  |  |  |
| H+ | C | 3 | 113 | 286.920 | 45.241 | 4.256 | 8.433 | 1966.975 | 783.150 | 73.673 | 145.973 | 1507.152 | 292.735 | 27.538 | 54.563 |  |  |  |  |
| H+ | H | -1 | 70 | 279.435 | 37.265 | 4.454 | 8.885 | 2240.502 | 620.204 | 74.129 | 147.882 | 1575.042 | 244.688 | 29.246 | 58.344 |  |  |  |  |
| H+ | H | 0 | 142 | 282.495 | 39.513 | 3.316 | 6.555 | 2087.726 | 738.101 | 61.940 | 122.451 | 1555.213 | 265.097 | 22.246 | 43.980 |  |  |  |  |
| H+ | H | 1 | 247 | 285.056 | 39.453 | 2.510 | 4.944 | 2240.406 | 729.732 | 46.432 | 91.454 | 1596.026 | 282.566 | 17.979 | 35.413 |  |  |  |  |
| H+ | H | 2 | 113 | 287.465 | 44.640 | 4.199 | 8.320 | 2214.684 | 774.470 | 72.856 | 144.355 | 1594.982 | 307.845 | 28.960 | 57.380 |  |  |  |  |
| H+ | H | 3 | 136 | 280.155 | 34.604 | 2.967 | 5.868 | 2203.555 | 737.813 | 63.267 | 125.122 | 1581.890 | 276.006 | 23.667 | 46.807 |  |  |  |  |
|  |  |  |  |  |  |  |  |  |  |  |  |  |  |  |  |  |  |  |  |
|  |  |  |  | **Sh** | | | | **Sfm** | | | | **Entropy** | | | |  |  |  |  |
| **Treatment** | **Partner** | **Phase of trial** | **N** | **mean** | **sd** | **se** | **ci** | **mean** | **sd** | **se** | **ci** | **mean** | **sd** | **se** | **ci** |  |  |  |  |
| H | C | -1 | 56 | 0.790 | 0.059 | 0.008 | 0.016 | 0.503 | 0.101 | 0.014 | 0.027 | 0.600 | 0.045 | 0.006 | 0.012 |  |  |  |  |
| H | C | 0 | 86 | 0.772 | 0.051 | 0.006 | 0.011 | 0.462 | 0.083 | 0.009 | 0.018 | 0.579 | 0.039 | 0.004 | 0.008 |  |  |  |  |
| H | C | 1 | 158 | 0.777 | 0.051 | 0.004 | 0.008 | 0.479 | 0.089 | 0.007 | 0.014 | 0.593 | 0.039 | 0.003 | 0.006 |  |  |  |  |
| H | C | 2 | 94 | 0.760 | 0.050 | 0.005 | 0.010 | 0.452 | 0.080 | 0.008 | 0.016 | 0.585 | 0.035 | 0.004 | 0.007 |  |  |  |  |
| H | C | 3 | 107 | 0.764 | 0.059 | 0.006 | 0.011 | 0.456 | 0.091 | 0.009 | 0.017 | 0.587 | 0.045 | 0.004 | 0.009 |  |  |  |  |
| H | H | -1 | 63 | 0.794 | 0.052 | 0.007 | 0.013 | 0.509 | 0.088 | 0.011 | 0.022 | 0.603 | 0.042 | 0.005 | 0.011 |  |  |  |  |
| H | H | 0 | 78 | 0.782 | 0.056 | 0.006 | 0.013 | 0.491 | 0.092 | 0.010 | 0.021 | 0.600 | 0.043 | 0.005 | 0.010 |  |  |  |  |
| H | H | 1 | 183 | 0.778 | 0.053 | 0.004 | 0.008 | 0.480 | 0.088 | 0.007 | 0.013 | 0.596 | 0.041 | 0.003 | 0.006 |  |  |  |  |
| H | H | 2 | 81 | 0.785 | 0.054 | 0.006 | 0.012 | 0.488 | 0.090 | 0.010 | 0.020 | 0.604 | 0.040 | 0.004 | 0.009 |  |  |  |  |
| H | H | 3 | 83 | 0.766 | 0.056 | 0.006 | 0.012 | 0.458 | 0.087 | 0.010 | 0.019 | 0.591 | 0.044 | 0.005 | 0.010 |  |  |  |  |
| H+ | C | -1 | 82 | 0.759 | 0.049 | 0.005 | 0.011 | 0.447 | 0.080 | 0.009 | 0.018 | 0.575 | 0.036 | 0.004 | 0.008 |  |  |  |  |
| H+ | C | 0 | 139 | 0.770 | 0.045 | 0.004 | 0.008 | 0.464 | 0.075 | 0.006 | 0.013 | 0.580 | 0.039 | 0.003 | 0.007 |  |  |  |  |
| H+ | C | 1 | 146 | 0.775 | 0.049 | 0.004 | 0.008 | 0.475 | 0.084 | 0.007 | 0.014 | 0.592 | 0.038 | 0.003 | 0.006 |  |  |  |  |
| H+ | C | 2 | 93 | 0.762 | 0.049 | 0.005 | 0.010 | 0.452 | 0.075 | 0.008 | 0.016 | 0.583 | 0.039 | 0.004 | 0.008 |  |  |  |  |
| H+ | C | 3 | 113 | 0.761 | 0.057 | 0.005 | 0.011 | 0.451 | 0.088 | 0.008 | 0.016 | 0.579 | 0.043 | 0.004 | 0.008 |  |  |  |  |
| H+ | H | -1 | 70 | 0.772 | 0.045 | 0.005 | 0.011 | 0.472 | 0.076 | 0.009 | 0.018 | 0.584 | 0.033 | 0.004 | 0.008 |  |  |  |  |
| H+ | H | 0 | 142 | 0.768 | 0.054 | 0.005 | 0.009 | 0.466 | 0.082 | 0.007 | 0.014 | 0.588 | 0.039 | 0.003 | 0.007 |  |  |  |  |
| H+ | H | 1 | 247 | 0.776 | 0.051 | 0.003 | 0.006 | 0.479 | 0.085 | 0.005 | 0.011 | 0.593 | 0.040 | 0.003 | 0.005 |  |  |  |  |
| H+ | H | 2 | 113 | 0.782 | 0.056 | 0.005 | 0.010 | 0.481 | 0.094 | 0.009 | 0.017 | 0.598 | 0.044 | 0.004 | 0.008 |  |  |  |  |
| H+ | H | 3 | 136 | 0.776 | 0.050 | 0.004 | 0.009 | 0.475 | 0.083 | 0.007 | 0.014 | 0.591 | 0.041 | 0.004 | 0.007 |  |  |  |  |

References

1 Tallet, C. *et al.* Behavioural and physiological reactions of piglets to gentle tactile interactions vary according to their previous experience with humans. *Livestock Sci* **167**, 331-341, doi:10.1016/j.livsci.2014.06.025 (2014).

2 Bensoussan, S., Tigeot, R., Meunier-Salaün, M.-C. & Tallet, C. Broadcasting human voice to piglets (Sus scrofa domestica) modifies their behavioural reaction to human presence in the home pen and in arena tests. *App. Anim. Behav. Sci.*, 104965, doi:https://doi.org/10.1016/j.applanim.2020.104965 (2020).

3 Friel, M., Kunc, H. P., Griffin, K., Asher, L. & Collins, L. M. Positive and negative contexts predict duration of pig vocalisations. *Scientific Reports* **9**, doi:10.1038/s41598-019-38514-w (2019).

4 Briefer, E. F., Vizier, E., Gygax, L. & Hillmann, E. Expression of emotional valence in pig closed-mouth grunts: Involvement of both source- and filter-related parameters. *Journal of the Acoustical Society of America* **145**, 2895-2908, doi:10.1121/1.5100612 (2019).

5 Syrova, M., Policht, R., Linhart, P. & Spinka, M. Ontogeny of individual and litter identity signaling in grunts of piglets. *Journal of the Acoustical Society of America* **142**, 3116-3121, doi:10.1121/1.5010330 (2017).
